# Mechanisms of Resistance to NK Cell Immunotherapy

**DOI:** 10.3390/cancers12040893

**Published:** 2020-04-07

**Authors:** Christian Sordo-Bahamonde, Massimo Vitale, Seila Lorenzo-Herrero, Alejandro López-Soto, Segundo Gonzalez

**Affiliations:** 1 Department of Functional Biology, Immunology, University of Oviedo, 33006 Oviedo, Spain; christiansbl87@gmail.com (C.S.-B.); seilalorenzoherrero@gmail.com (S.L.-H.); 2 Instituto Universitario de Oncología del Principado de Asturias, IUOPA, 33006 Oviedo, Spain; lopezsalejandro@uniovi.es; 3 Instituto de Investigación Biosanitaria del Principado de Asturias (ISPA), 33011 Oviedo, Spain; 4 UO Immunologia, IRCCS Ospedale Policlinico San Martino Genova, 16132 Genoa, Italy; massimo.vitale@hsanmartino.it; 5 Department of Biochemistry and Molecular Biology, University of Oviedo, 33006 Oviedo, Spain

**Keywords:** cancer, immunotherapy, NK cell, CAR-NK, HSCT, ADCC, adoptive transfer, resistance, immunoediting, immunosuppression, microenvironment

## Abstract

Immunotherapy has recently been a major breakthrough in cancer treatment. Natural killer (NK) cells are suitable targets for immunotherapy owing to their potent cytotoxic activity that may target cancer cells in a major histocompatibility complex (MHC) and antigen-unrestricted manner. Current therapies targeting NK cells include monoclonal antibodies that promote NK cell antibody-dependent cell-mediated cytotoxicity (ADCC), hematopoietic stem cell transplantation (HSCT), the adoptive transfer of NK cells, the redirection of NK cells using chimeric antigen receptor (CAR)-NK cells and the use of cytokines and immunostimulatory drugs to boost the anti-tumor activity of NK cells. Despite some encouraging clinical results, patients receiving these therapies frequently develop resistance, and a myriad of mechanisms of resistance affecting both the immune system and cancer cells have been reported. A first contributing factor that modulates the efficacy of the NK cell therapy is the genetic profile of the individual, which regulates all aspects of NK cell biology. Additionally, the resistance of cancer cells to apoptosis and the immunoediting of cancer cells, a process that decreases their immunogenicity and promotes immunosuppression, are major determinants of the resistance to NK cell therapy. Consequently, the efficacy of NK cell anti-tumor therapy is specific to each patient and disease. The elucidation of such immunosubversive mechanisms is crucial to developing new procedures and therapeutic strategies to fully harness the anti-tumor potential of NK cells.

## 1. Natural Killer (NK) Cells

Natural killer (NK) cells are innate lymphoid cells that play a key role in the innate response to viral infection and cancer [1,2]. Unlike T cells, NK cells distinguish viral-infected and cancer cells from their healthy counterparts through an array of germline-encoded activating and inhibitory receptors. Inhibitory receptors prevent NK cell activation and the subsequent killing of healthy cells via binding to surface self-proteins such as ubiquitously expressed major histocompatibility complex class I molecules (MHC-I). The loss of surface expression of these molecules, which is frequently caused by viral infection or malignant transformation, results in “missing self recognition” by NK cells, and leads to NK cell activation and the killing of target cells [3]. Inhibitory receptors that recognize MHC class I molecules, such as Killer cell immunoglobulin-like receptors (KIRs) and the heterodimer CD94-Natural Killer Group 2A (NKG2A), have been more extensively studied [4,5,6]. Programmed cell death 1 (PDCD1, best known as PD-1) and other immune checkpoints receptors may inhibit NK cell activation, particularly in viral infection and cancer [6,7,8,9]. Activating receptors, including Natural killer group 2D (NKG2D), DNAX accessory molecule-1 (DNAM-1) and the natural cytotoxicity receptors NKp46, NKp44 and NKp30, recognize inducible ligands that are specifically upregulated on infected, cancer and stressed cells [4,5,10,11,12]. NKG2D is the most extensively studied NK cell activating receptor and binds to a group of stress-inducible ligands termed MHC class I polypeptide-related sequence A and B (MICA and MICB) and UL16 binding proteins (ULBP1-6) [10,11]. NKG2D ligands are restrictedly expressed in healthy cells, but their expression is induced in response to signaling pathways commonly associated with transformation and viral infection. Thus, DNA damage, oxidative stress or proliferative stress signaling pathways induce the expression of NKG2D ligands, initiating an immune response against the target cells [10,13,14]. A positive balance of signals provided by activating and inhibitory receptors promotes NK cell activation, resulting in target cell elimination via the exocytosis of cytotoxic granules containing perforin and granzymes. Target cells may also be killed by a second pathway involving Fas ligand (FasL) and tumor necrosis factor (TNF)-related apoptosis-inducing signals [4]. Additionally, human NK cells express FcγRIIIa receptor (also known as CD16), which recognizes IgG_1_ and IgG_3_ human antibodies, hence allowing the NK cell-mediated elimination of IgG-opsonized cells through the release of their cytotoxic granules, a process referred to as antibody-dependent cellular cytotoxicity (ADCC). ADCC has been shown to contribute to defense against viral infection and the therapeutic efficacy of some monoclonal antibodies (mAbs) widely used to treat cancer, such as rituximab. It has also been reported that some immune checkpoint inhibitors, such as ipilimumab, may also trigger ADCC [5,12,15,16]. The activity of NK cells is regulated by type I interferons and cytokines (see below), and NK cells also regulate the innate and adaptive immune responses through the secretion of cytokines, such as interferon-gamma (IFN-γ), with potent anti-viral and anti-tumor activities [4]. However, the poor infiltration of NK cells into solid tumors, changes in activating/inhibitory signaling and the tumor microenvironment decrease the NK-mediated killing of malignant cells. 

## 2. NK Cell-Based Therapies

Immunotherapy has been a recent major breakthrough in cancer treatment. Despite most of the current cancer immunotherapies being focused on T cells, NK cells are being increasingly considered to be a key target of immunotherapy (as recently reviewed in [17,18]) (Table 1). For example, their ADCC function can contribute to the cytotoxic effects of different mAbs used in the therapy of many cancers, especially, but not limited to, those of hematological origin [18,19,20,21]. Rituximab (a mAb specific to the B cell marker CD20), trastuzumab (anti-ErbB2/HER2 mAb) and cetuximab (an anti-EGFR mAb) have proved marked efficacies in the treatment of various solid and hematological tumors [22]. In fact, more than a million patients have been treated with rituximab over two decades. Several mechanisms of action account for the clinical activity of these cytotoxic mAbs, including ADCC, the direct apoptosis of tumor cells and complement-dependent cytotoxicity (CDC) [23]. The relative contribution of these mechanisms of action to the therapeutic efficacy of rituximab and other mAbs has not been fully established, although ADCC is thought to be the main mechanism underlying their efficacies [24]. Recently, a new generation of NK cell-activating antibodies has been developed [25]. Particularly, bispecific and trispecific killer cell engagers (BiKEs and TRiKEs) that stimulate NK cells against one or more tumor antigens are promising agents that are in preclinical and initial clinical studies [25,26,27]. Similarly, novel mAbs that block NK cell-expressed checkpoint proteins and inhibitory receptors—including PD-1/PD-L1, NKG2A-HLA-E, Tim-3, LAG-3, TIGIT and CD96—are under preclinical and clinical evaluation [8,18,28,29,30,31,32,33].

The therapeutic effect of hematopoietic stem cell transplantation (HSCT) mainly relies on the allogenic immune response against the cancer cells exerted by the donor’s T and NK cells [34]. Outstanding clinical responses are observed in patients with acute myeloid leukemia (AML) upon transplantation from KIR/MHC class I mismatched donors, hence evidencing that HSCT may fully unleash the anti-tumor potential of NK cells [35]. HSCT may be refined by the direct adoptive transfer of autologous or allogenic NK cells [18]. The redirection of NK cells using chimeric antigen receptor (CAR)-NK cells is another alternative for boosting NK cell therapeutic efficacy. CAR-NK cells targeting several types of tumors, employing both primary NK cells or NK-92 cell line as carriers, are currently being investigated in preclinical and initial clinical trials [36]. 

The anti-tumor activity of NK cells may be potentiated by cytokines, particularly IL-2, which was initially considered to be a promising anti-neoplastic drug for its capacity to boost T cell and NK cell anti-tumor activity [37]. Unfortunately, its toxicity, the IL-2-driven stimulation of regulatory T cells (Tregs) and its limited efficacy have restricted the clinical use of this cytokine in tumor immunotherapies, and efforts have been made to improve its efficacy by combining it with other anti-cancer regimens and therapies [37]. Cytokines that activate NK cells without stimulating Treg cells—including IL-12, IL-15, IL-18 and IL-21—have great potential to be harnessed in cancer therapy [38]. In particularly, IL-12 and IL-21 have demonstrated great potential to increase ADCC-mediated killing by NK cells in solid tumors [39,40]. IL-15 is a cytokine that, like IL-2, strongly activates both NK cells and CD8 T cells, but inducing less immunosuppression and toxicity [41]. Initial clinical trials involving the administration of IL-15 in monotherapy or in combination with NK cells or chemotherapy in patients with hematological and solid tumors are currently ongoing. These include the IL-15 receptor agonist ALT-803 which has recently shown encouraging clinical results in advanced solid tumors in a phase I clinical trial (NCT01727076). Clinical trials using recombinant IL-15 in combination with, for example, CAR-NK/T cell, checkpoint blockade and haploidentical donor NK cell infusion-based therapies, are currently ongoing (source: http://clinicaltrials.org) [42,43]. 

The immunomodulatory drugs (IMiDs) lenalidomide and pomalidomide display both direct anti-neoplastic activity on hematological cancer cells and a modulatory effect on multiple immune cell types, including NK cells [44,45]. Despite the fact that the real contribution of such different mechanisms to the therapeutic activity of these drugs remains to be fully established, the role of NK cells appears to be relevant [45,46]. Indeed, lenalidomide markedly increases NK cell activation and proliferation through the induction of IL-2 production by CD4 T cells in chronic lymphocytic leukemia (CLL) [46,47]. Also, lenalidomide increases NK cell numbers, promotes the expression of activating receptors, such as CD16, and reduces that of checkpoint receptors, thus enhancing NK cell-mediated cytotoxicity and ADCC [45,46,48,49,50]. Moreover, lenalidomide increases the expression of NKG2D and DNAM-1 ligands (MICA and PVR) in multiple myeloma [51]. These effects support the combination of IMiDs with cytotoxic mAbs, such as rituximab, as a potential therapeutic strategy to be harnessed. Noteworthily, a number of anti-neoplastic molecules that possibly influence NK cell activation or NK–tumor cell interactions have been proposed in these latter years, so elucidating the possible synergistic effects of anti-neoplastic drugs and NK cells currently represents an interesting field of investigation [52].

## 3. Are NK Cells Suitable Targets for Immunotherapy?

NK cells should be tested as a potential alternative for cancer immunotherapy owing to their potent cytotoxic activity that may target cancer cells in an MHC- and antigen-unrestricted manner. Thereby, they can be used as donors for immunotherapy [53]. Furthermore, the expression of CD16 on NK cells supports their combination with ADCC-promoting mAbs. Along with this potent anti-tumor activity, NK cells may have some potential advantages over T cell-based therapies. Thus, the so-called “cytokine storm” or “cytokine release syndrome” associated with excessive T cell activation may be fatal, and it is largely mediated by the production of pro-inflammatory cytokines by T cells. Conversely, NK cells produce cytokines, including IFN-γ and GM-CSF, with lower toxicity profiles than those produced by T cells [53]. NK cells are short lifespan cells, and NK cell-based therapies, such as those using CAR-NK cells, are not expected to be associated with long-term problems, such as the risks of autoimmunity or malignant transformation that are characteristic of CAR-T cells [53]. In a similar line of evidence, a major complication of HSCT is the development of the so-called graft-versus-host disease (GvHD) that is caused by allogenic T cells. NK cells recover quickly after HSCT and do not cause GvHD, representing an attractive means to improve therapeutic efficacy without increasing toxicity [54,55]. However, NK cells have some drawbacks as targets for immunotherapy. Despite some encouraging reports of clinical efficacy, infused NK cells showed limited expansion, persistence and capability of infiltrating solid tumors in vivo, which are major determinants of the clinical response [17,18,56,57]. In order to enhance their expansion and activation, NK cells need to be pre-activated with cytokines or feeder cells (Table 1) [58]. For instance, clinical responses have been improved by the pre-activation of NK cells with IL-12, IL-15 and IL-18 to generate the so-called cytokine-induced memory-like (CIML) cells [59]. Following the same line, cytokine-induced killer (CIK) cells have been generated by the ex vivo expansion of peripheral blood mononuclear cells (PBMCs) with cytokines, resulting in cells with features mixed between those of cytotoxic T cells and NK cells [60]. CAR-NK cells also show a low persistence, cytotoxicity and capacity to infiltrate tumors in vivo. To improve proliferation and persistence, several strategies are being pursued, including the development of CAR-NK cells expressing IL-15, which are under clinical evaluation (NCT03056339). Alternatively, NK cell-derived cell lines, such as NK-92, have been studied as a source of highly active NK cells. The use of CAR NK-92 cells may have certain advantages, such as their lower production cost when compared with CAR-T cells (under good manufacturing practices conditions), their high cytotoxic activity against a broad spectrum of malignant cells, their ADCC-mediated killing capability (only in the NK92 cell line previously transfected with the gene encoding CD16) and their lack of inhibitory receptors [61,62]. Nevertheless, there are some limitations that need to be addressed to make them more suitable for anti-cancer therapy. CAR NK-92 cells have to be irradiated to prevent their expansion, inducing a short life, decreased anti-tumor activity and IFN-γ production, and a lack of certain inhibitory KIRs that may induce GvHD [63,64,65]. Finally, NK cells are difficult to obtain, expand and manipulate, as they show rather low transfection efficiencies, even when viral vectors are used [66]. Overall, NK cells are promising targets for immunotherapy; however, many issues regarding the procedures for manipulation, activation and expansion need to be addressed to fully harness their anti-tumor potential.

## 4. Resistance to NK Cell Therapy

**Figure 1:**
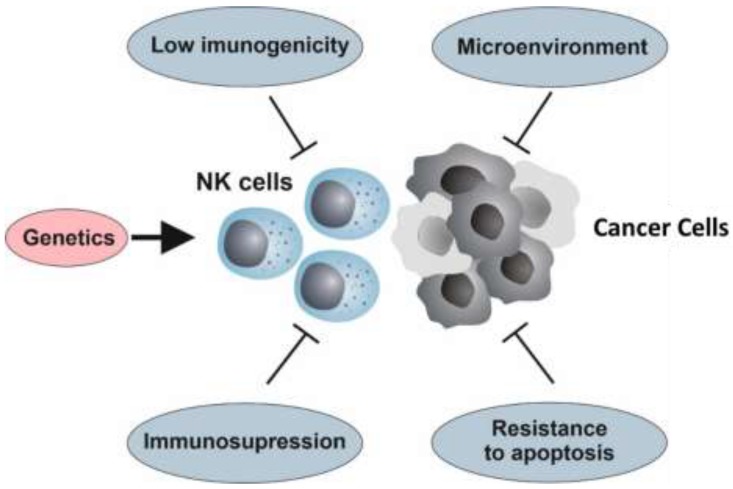
Resistance to natural killer (NK) cell-mediated immunotherapy. The genetic background that modulates the biology of NK cells, the resistance of cancer cells to apoptosis, and the complex interaction between the tumor and its microenvironment with the immune system—a process known as immunoediting, which may reduce the cancer’s immunogenicity, thus promoting immunosuppression—are crucial factors of tumor resistance to NK cell-based therapies.

Resistance may be defined as a lack of response to therapy (intrinsic resistance) or as disease progression after an initial clinical benefit (acquired resistance). The incidence of resistance is difficult to evaluate and may vary significantly depending on its definition [67]. Nevertheless, it has been roughly established that 30–60% of patients with indolent non-Hodgkin lymphoma (NHL) are resistant to rituximab, 70% of patients with HER2-positive breast cancers show intrinsic or secondary resistance to trastuzumab and only 15–20% of patients with head and neck cancer respond to cetuximab [67,68]. Despite being investigated for over two decades, the mechanisms of tumor resistance to rituximab remain poorly understood. Nevertheless, mechanisms of resistance that hamper the three major pathways of rituximab’s action―ADCC, CDC and the induction of apoptosis―have been reported [67]. Similarly, resistance to cetuximab has been reported in colorectal cancer and head and neck cancer, causing low objective response rates Primary and acquired resistance have been associated with many aspects of cetuximab’s biology, including receptor internalization, genetic polymorphisms in the EGFR gene and mutations in the KRAS gene, affecting both the ability of cetuximab to block EGFR-mediated signal transduction and ADCC [69,70]. Further, mAbs share resistance mechanisms with other anti-cancer drugs, including altered pharmacokinetics, deregulated metabolism and reduced diffusion into the tumor site. Nevertheless, in this review, we only focus on those mechanisms specifically involved in cancer resistance to the NK cell-mediated immune response (Figure 1). Overall, the genetic background of the individual, which modulates the biology of NK cells; the resistance of cancer cells to apoptosis; and a complex interaction between cancer cells and the immune system, which limits the cancer’s immunogenicity and promotes immunosuppression—are major determinants of the resistance to/efficacy of NK cell therapy. 

### 4.1. The Genetic Background 

Despite the ongoing discovery of genetic variants associated with immunodeficiency and susceptibility to autoimmune disease and infection, the genetic control of the human immune system, particularly under homeostatic conditions, remains poorly understood [71,72]. Genetic approaches have led to the identification of genes that modulate NK cell development, recognition and cytotoxicity, which are likely to influence NK cell therapy [71,72]. For instance, the proportion of NK cells varies between individuals, representing 5–15% of circulating lymphoid cells, and such variation is, in part, heritable [73,74]. Genome-wide association studies (GWAS) and functional studies have demonstrated the association between a 17q12 allele (rs9916629C) with a lower level of NK cells in the peripheral blood, an altered distribution of NK cell subsets with a lower proportion of cytotoxic CD56^dim^ NK cells, and altered ADCC [73,75]. A limited cytotoxic activity of peripheral blood lymphocytes has been associated with an increased cancer risk in an 11-year follow-up study [76], and reduced NK cell numbers and function have also been associated with poorer outcomes in patients with follicular lymphoma (FL) and diffuse large B cell lymphoma (DLBCL) that are receiving anti-CD20 based immunotherapies [77]. Furthermore, low levels of infiltrating NK cells have been correlated with local recurrence in patients with colorectal cancer, as well as with the response to treatment and prognosis in breast cancer [78,79,80]. Similarly, a decreased CD16 expression on NK cells and an impaired ADCC activity in patients with DLBCL at diagnosis were also observed [81]. Nevertheless, in spite of the fact that low NK cell numbers and activity have been associated with a hampered efficacy of NK cell-based therapies [17,18], the genetic influence in this scenario remains to be established.

Some genetic polymorphisms involved in the regulation of NK cell functions have been associated with the efficacy of NK cell therapies. A nucleotide substitution in the *FCGR3A* gene (the gene encoding FcγRIIIa receptor) (rs396991; T > G) results in valine or phenylalanine expression at amino acid 158 (V158F). Individuals with valine at position 158 show a higher affinity for rituximab, an augmented NK cell-mediated ADCC activity [82] and better clinical responses to rituximab in FL [83,84], Waldenström macroglobulinemia [85], and DLBCL patientstreated with rituximab plus CHOP (R-CHOP) [86]; although the presence of valine at position 158 does not predict clinical responses in CLL [87]. Furthermore, *FCGR3A* polymorphisms are involved in CD16 transcription and protein expression, hence influencing NK cell-mediated ADCC [82,88]. *FCGR3A* (V158F) polymorphism exerts similar effects on the effectiveness of trastuzumab-based therapies [89,90] and cetuximab-mediated ADCC, as well as other cetuximab-based therapies [91,92]. These observations have led to the development of novel glycol-engineered humanized anti-CD20 antibodies, such as obinutuzumab, with higher affinities for CD16 [93]. Preclinical and clinical data have shown an increased anti-tumor efficacy of obinutuzumab compared to that of rituximab in CLL and indolent lymphomas, albeit clinical data are still preliminary and based on higher doses of obinutuzumab [23,94].

In the context of HSCT, clinical experience shows that allogenic NK cells are more efficacious than autologous NK cells, as they may take advantage of the “missing-self” recognition due to the lack of KIR-HLA class I interaction. *KIR* genes are highly polygenic and polymorphic, with different numbers of activating and inhibitory *KIR* genes in different individuals, each of them varying in expression and functional activity [95]. *KIR* (chromosome 19) and *HLA class I* (chromosome 6) genes segregate independently, which results in variable KIR-HLA class I combinations in individuals. To establish self-tolerance, the inhibitory KIRs and HLA class I alleles of each individual constitute the NK cell repertoire during development. Thus, the engagement of inhibitory KIRs with self HLA-I molecules educates or “licenses” NK cells for function [95]. KIR-HLA class I combinations significantly affect NK cell activity, ADCC and prognosis [96,97], and they have a significant impact on infection outcomes and autoimmune disease susceptibility [72]. In the same line of evidence, *KIR* polymorphisms and HLA class I alleles have been shown to be involved in the efficacy of anti-GD2 mAb-based therapy in children with high-risk neuroblastomas [96,98] and in the efficacy of rituximab in FL [99]. Notably, the influence of KIR/HLA interactions has been shown to act in synergy with the effect of *FCGR3A* (V158F) single nucleotide polymorphisms in NK cells [100]. *KIR* and HLA class I genes also play a key role in the efficacy of HSCT. Patients with AML lacking KIR ligands in T cell-depleted haploidentical donors exhibited a reduced risk of leukemia relapse and GvHD, as well as increased overall survival [54,55]. Contrarily, adverse effects of a KIR–ligand mismatch on overall survival in haploidentical HSCT and cord-blood transplants were observed [101,102]. Specific KIR activating genes, such as *KIR3DS1*, have also been associated with less GvHD in allogeneic HSCT [103], whereas others, such as *KIR2DS1*, protect from leukemia relapse [104,105]. 

Despite the above-mentioned evidence, data regarding the genetic architecture that modulates NK cell life and function, and, especially, their implication in the efficacy of/resistance to NK cell-based therapies, are scarce. Some pieces of information clearly suggest that the genetic background of the individual may be an intrinsic determinant for the degree of NK cell therapy success. This matter clearly warrants further investigation.

### 4.2. Hallmarks of Cancer and Resistance to NK Cell Immunotherapy

In a seminal article, Hanahan and Weinberg defined common traits ("hallmarks") that govern the transformation of normal cells to tumorigenic cells [106]. Some of these intrinsic hallmarks, especially the resistance to apoptosis, have a significant impact on the success of anti-cancer chemotherapies and immunotherapies. Practically all cancer cells inactivate key components of the apoptotic machinery, which allows them to survive under a variety of cell stressors including hypoxia, signaling imbalances, DNA damage and a loss of anchorage [106]. Inactivation of the p53 pathway or other pro-apoptotic pathways, along with the activation of pro-survival oncogenic and anti-apoptotic pathways, deeply impacts the efficacy of chemotherapy. For instance, the overexpression of anti-apoptotic members of the Bcl-2 family is frequent in B cell NHL and predicts aggressive disease, chemotherapy resistance and a poorer prognosis [107]. A overlap between resistance to chemotherapy and resistance to rituximab has been reported, suggesting common underlying mechanisms [108]. Similarly, cell lines resistant to rituximab, trastuzumab and cetuximab have been generated in vitro by continuous long-term exposure to these drugs, showing a hyper-activation of pro-survival and anti-apoptotic proteins or a deficiency in the expression of pro-apoptotic proteins [109,110,111,112,113,114,115,116]. Interestingly, resistance may be overcome with inhibitors of these survival pathways [112].

The overexpression of X-linked inhibitor of apoptosis (XIAP), a potent caspase inhibitor member of the inhibitor of apoptosis proteins (IAP) family, is associated with aggressiveness and resistance to chemotherapy and targeted therapy in breast cancer and other malignancies [117]. Its overexpression also drives the resistance to ADCC mediated by cetuximab and trastuzumab [118]. These pro-apoptotic proteins are attractive targets to reduce resistance to NK cell therapies. Thus, the neutralization of IAP proteins by the overexpression of the Second Mitochondria-derived Activator of Caspases (SMAC) fusion protein greatly enhanced the susceptibility of tumor cells to killing by lymphokine-activated killer (LAK) cells [119]. Similarly, resistance to NK cell killing may be overcome by using SMAC mimetics, a new class of targeted drugs that act as IAP antagonists. SMAC mimetics increase NK cell proliferation in vitro and in vivo [120], increase cytotoxicity [121,122,123,124] and overcome rituximab resistance in preclinical models of B cell lymphoma [125]. Furthermore, SMAC mimetics sensitize sarcomas and hematological cancers to CIK cell adoptive therapy [126,127] and they have also demonstrated clinical efficacy in mouse models of glioblastoma and osteosarcoma, alone or in combination with checkpoint blockade [128,129]. 

Tumors are more than cancer cells; they are complex tissues that depend on a myriad of heterotypic interactions, with multiple cell types present in the tumor microenvironment. At the biochemical level, such an interdependence is manifested by the exchange of several oncogenic and trophic factors. These survival and anti-apoptotic signals from the microenvironment may also be critical regulators of therapy effectiveness. This is exemplified by the protection of CLL leukemia cells from rituximab-induced apoptosis in vitro provided by their direct contact with stromal cells [130]. In vivo, B cells from blood appear to be more easily depleted by rituximab than B cells present in the lymph nodes and bone marrow [67]. Moreover, B cells in the germinal centers of lymph nodes receive survival signals, rendering them more resistant to rituximab. In fact, NK cells are more efficient at eliminating blood cancer cells, small tumors and metastases than large solid tumors [17,18,57]. This clearly means that the tumor microenvironment is a major barrier to the success of NK cell anti-tumor therapies. In most studies, low numbers and impaired function were observed in NK cells isolated from the primary tumor [57]. This is mainly due to the accumulation of suppressive factors in the tumor microenvironment, which dampens NK cell anti-tumor activity and reduces the recruitment of NK cells into the tumor nest and their persistence [57] (see below). A more profound knowledge of the complex interactions between NK cells and the tumor microenvironment in solid tumors will open new vistas for cancer therapy. 

### 4.3. Immunoediting

Cancer immunosurveillance sculpts the immunogenic phenotype of tumors as they develop, leading to the development of cancer cells capable of evading the immune response [131]. This process, referred to as immunoediting, encompasses three phases that are termed elimination, equilibrium and escape. Throughout these phases, the immunogenicity of the tumor is reduced and immunosuppressive mechanisms are acquired, both contributing to immune evasion [131,132]. Evidence of NK cell immunoediting have been obtained from knockout mice lacking NKG2D, DNAM-1 and NKp46, which developed tumors with a higher expression of ligands of these activating receptors [133,134,135]. NK cell immunoediting has even been evidenced in the absence of an adaptive immune system [136]. There are a myriad of pieces of evidence showing that NK cells also sculpt the cancer phenotype in humans, which is particularly evident in the loss of ligands of NK cell activating receptors and the upregulation of inhibitory molecules in cancer cells [137]. For instance, decreased NKG2D ligand expression, in particular, a reduction of MICA surface levels by proteolytic shedding, has been widely described and associated with advanced disease and a poor prognosis in different types of cancers [10,11,138,139]. MICA expression was also shown to be an independent prognostic factor for survival in patients with gastric cancer who received adjuvant therapy combined with CIK [140]. These data clearly suggest that cancer development in an immunocompetent individual generates tumors with a certain degree of intrinsic resistance to NK cell-mediated therapy. Consequently, treatments that revert these mechanisms of immune evasion may potentiate NK cell therapy. For instance, all-trans retinoic acid (ATRA) upregulates MICA expression when combined with CIK, enhancing the activity of cytotoxic lymphocytes in patients with lung adenocarcinoma [141]. 

Despite of there are robust evidences about the effect of the immunoediting process on cancer cells, these cells also affect NK cells inducing a progressive loss of their cytotoxic capability and IFN-γ secretion [142,143]. In particular, cancer stem cells (CSCs) may play a significant role in this process. CSCs represent a subpopulation of tumor cells highly resistant to chemotherapy and radiotherapy that have been classically thought to be susceptible to NK-cell mediated killing due to their low MHC class I expression [144,145]. Accordingly, the efficacy of the NK cell targeting of CSCs has been demonstrated in several solid tumors in experimental models, including of colorectal cancer and melanoma [146,147,148]. However, secretion of suppressor cytokines orshedding of soluble NKG2D ligands by CSCs may play a key role in decreasing NK cell-mediated cytotoxicity and IFN-γ secretion [149,150].

The immunoediting process is also evident in the reduction of MHC class I molecules frequently observed in cancer cells that thereby prevents antigen recognition by T cells [151]. To further avoid recognition by NK cells, cancer cells frequently upregulate the expression of the non-classical MHC class I molecules HLA-E (which binds to the inhibitory receptor NKG2A/CD94) and HLA-G (which binds, at least, to the inhibitory receptors ILT2, KIR2DL4 and CD160), mediating self-tolerance and inducing resistance to NK cell therapies [49,152,153,154]. Furthermore, the NKG2A-HLA-E system plays a central role in NK cell-mediated immune evasion after allo-HSCT [153,155]. In this context, monalizumab, a mAb that blocks NKG2A-HLA-E interaction, shows potent anti-tumor activity and may significantly enhance the NK cell-mediated graft vs. leukemia (GvL) effect [33,156]. 

The loss or modulation of the target antigen as a consequence of the immunoediting process is one of the most obvious mechanisms of acquired resistance to NK cell-mediated ADCC. Thus, the downregulation of CD20 expression is a common mechanism of rituximab resistance [67] as observed in lymphoma cells and patients repeatedly exposed to rituximab [157,158,159]. The complete loss of CD20 after rituximab therapy is infrequent [67], but multiple alterations in CD20 expression or structure have been identified including, but not limited to, mutations in *MS4A1*, the gene encoding CD20 [159]; CD20 internalization [160]; conformational changes in the CD20/rituximab complex on the cell membrane [161]; the generation of tumor-derived exosomes that sequester antibodies and prevent ADCC [162]; and the removal of rituximab/CD20 complexes from the cell surface by monocytes through the Fc receptor [163]. In the same line of evidence, truncated forms of the HER2 receptor or the expression of other HER family members that enable alternative dimerization patterns have strongly been associated with resistance to trastuzumab [114,164,165]. The dysregulation of the surface expression of EFGR is also associated with resistance to cetuximab [111]. However, despite this accumulation of data, the underlying mechanisms remain to be established. 

To conclude, the immunoediting process exerted during cancer transformation and progression leads to the rise of cancer clones resistant to immune attack. Those mechanisms of immune evasion acquired during cancer development in an immunocompetent individual broadly provide intrinsic resistance to immunotherapy [166]. Immunotherapy additionally exerts a selection pressure on tumor cells, generating “escape mutants”, clearly exemplified by the loss or modulation of CD20 expression in patients under rituximab therapy, which may further lead to the development of acquired resistance.

### 4.4. Immunosuppression

In recent years, it has been well established that the development and progression of many malignancies are accompanied by immunosuppression, which often targets NK cells, both at the tumor site [2,167,168,169,170] and at the systemic level [2,167,171,172,173,174]. Tumor cells play a crucial role in this context, as they may both directly inhibit immune effector cell functions and contribute to the generation of a local suppressive microenvironment that hinders immune responses at the tumor site. The direct suppression of NK cells may be exerted by tumor cells in different ways, as follows. i) Cancer cells can release or express soluble immunomodulatory factors such as TGF-β, PGE_2_ and the enzyme Indoleamine 2,3-dioxygenase (IDO). TGF-β affects the metabolism of NK cells [175] and reduces NKG2D and NKp30 expression [176]. PGE2 inhibits NK cell differentiation, cytotoxicity and NKp44 and NKp30 expression, and may interfere with the NK cell-mediated recruitment of dendritic cells at the tumor site [177,178]. IDO plays an immunosuppressive role by causing both tryptophan (Trp) shortage and increased production of the Trp catabolite kynurenine, which downregulates NKp46 and NKG2D expression [28,179]. ii) Tumor cells may also express decoy ligands, capable of inhibiting the signaling of NK cell activating receptors, or even inducing their internalization from the NK cell surface. The soluble form of the extracellular matrix (ECM)-associated nidogen-1 glycoprotein (sNID1) and the β-galactoside binding protein galectin-3 have recently been described as decoy ligands for NKp44 and NKp30, respectively [180,181]. Ligand shedding from the tumor cell surface represents another source of decoy ligands. For example, the proteases ADAM10 and ADAM17, expressed at the surfaces of different tumor cell types, may cleave the extracellular domains of the NKG2D ligands, MICA and MICB, and that of the NKp30 ligand, B7-H6 [182,183]. iii) Tumor cells may suppress NK cells by expressing ligands for the so-called checkpoint inhibitory receptors, which are variably expressed by NK cells. PD-1 has been shown to be expressed in blood NK cells from patients with multiple myeloma, and in blood and tumor-associated NK cells from patients with renal and ovarian cancers [29,184,185]. TIGIT, TIM-3 and LAG-3 represent additional checkpoint receptors for NK cells. Remarkably, all of them appear to play a role in the induction of functional exhaustion, which often characterizes tumor-associated NK cells [186,187,188]. The cytokine-inducible SH2-containing protein (CIS), which controls IL-15 signaling in NK cells, has also been proposed as an intracellular checkpoint. Targeting the CIS encoding gene, *Cish*, resulted in an increased NK cell-dependent resistance to tumor initiation and metastases in mice. Moreover, combining Cish deficiency with targeted therapies or immune-control blockade therapies further improved the control of metastasis [189]. Finally, IL-1R8 represents an additional checkpoint endowed with anti-inflammatory properties, and its inhibition increased NK cell anti-tumor responses in mice and the production of IFN-γ by human NK cells [190].

Tumor cells may also induce several regulatory cells that inhibit NK cell function and, in solid tumors, also contribute to the generation of a suppressive microenvironment at the tumor nest. Tregs, myeloid-derived suppressor cells (MDSCs), tumor-associated macrophages (TAMs) and tumor-associated fibroblasts (TAFs) may inhibit NK cell function, either by releasing TGF-β, IL-4 and PGE2, or by contributing to the production of the suppressive factors kynurenine (via IDO) and adenosine (via CD39 and CD73) [191,192]. In addition, the tumor microenvironment is frequently characterized by hypoxia, a condition that profoundly influences NK cells by affecting their metabolism, cytokine production, and expression and function of several activating receptors [193,194,195]. Hypoxia may also indirectly target NK cells by favoring tumor cell transition into an NK cell-resistant phenotype and inducing PD-L1 expression on MDSC and tumor cells [196,197,198]. The NK cell response to hypoxia is mediated by hypoxia-inducible factors (HIFs), and targeting HIF-1a on NK cells has been shown to inhibit tumor growth in an animal model [199]. The low oxygen tension affects not only solid tumors, as the levels of hypoxia are also increased (and this increase plays a role)) in the bone marrow of patients with multiple myeloma or leukemia [200]. However, whether hypoxia could effectively influence NK cell-mediated anti-tumor activity in hematological malignancies has not yet clearly been investigated.

Another important issue related to the tumor microenvironment regards the recruitment of effective NK cells at the tumor core. Different studies on tumor specimens and tissue arrays have revealed that the NK cell infiltrate is often limited, comprising poorly cytotoxic cells, such as those displaying the CD56^bright^CD16^dim^ phenotype (hereafter referred as CD56^bright^ cells) [2,201]. In this context, the tumor microenvironment plays a role by influencing both the local chemokine milieu and the chemokine receptor expression on NK cells. Increased gene expression of chemokines attracting CD56^bright^ NK cells (CXCL9, CXCL10 and CCL19) has been shown in breast and lung tumor tissues, and it has been correlated with the presence of CD56^bright^ NK cells within the lymphocyte infiltrate [202,203]. Hypoxia may also influence the surface expression of CCR7 and CXCR4 on CD56^bright^ NK cells, increasing their migration response to CCL19/21 and CXCL12 [195]. CXCL12 is expressed by many tumor types and supports tumor survival, and, together with CCL19/21, also favors metastatic spread [204,205,206]. Thus, hypoxia may promote the recruitment of poorly cytotoxic CD56^bright^ NK cells to primary tumor nests or metastases. Similarly, neuroblastoma cells have been shown to upregulate CXCR4 and CXCR3 on both CD56^dim^ and CD56^bright^ NK cells and to reduce CX_3_CR1 (which is important for NK cell extravasation) on CD56^dim^ NK cells viarelease of TGF-β. Finally, neuroblastoma cells have been shown to upregulate CXCR4 and CXCR3 and reduce CX_3_CR1 expression on NK cells via the release of TGF-β [207]. In this case, the outcome of the overall chemokine receptor modulation is less clear-cut; however, the downregulation of CX_3_CR1 suggests that the recruitment of cytotoxic CD56^dim^ NK cells may be reduced in neuroblastoma lesions.

The most recent NK cell-based therapeutic approaches have been designed with the intent of overcoming or limiting many of these suppressive effects. However, this task still appears to be rather challenging, as a single tumor type may avoid the activity of NK cells by multiple, and often concurrent mechanisms, that are either redundant or affect different aspects of NK cell biology. Targeting tumor cells via NK cell-mediated ADCC is promising, as this function appears to be poorly influenced by hypoxia or TAFs, as both hypoxia and TAFs appear to have no influence on the expression and function of CD16 in vitro [178,194,208]. However, other suppressive mechanisms may limit its efficacy. For example, TGF-β can inhibit NK cell-mediated IFN-γ production and ADCC [209]. In this regard, its role in inducing CD16 downregulation and inhibiting of cetuximab-mediated ADCC has been reported in patients with esophageal squamous cell carcinoma [210,211,212]. Complement activation may represent an additional mechanism of resistance, asdepletion of the C3 component increased NK cell-mediated ADCC against rituximab-coated target cells and improved the efficacy of antibody therapy in an in vivo model [213]. Further, NK cells from patients treated with rituximab showed a CD16 downregulation that could last up to 48 hours post infusion [214]. In line with this observation, CD16 stimulation may induce either receptor internalization or shedding on NK cells via ADAM17 [214,215]. Along these lines, strategies targeting tumor ADAMs may synergize with ADCC, although it is still debated whether ADAM17-mediated CD16 cleavage could induce NK cell detachment from the target cell and eventually favor tumor escape. NK cell-mediated ADCC may also be dampened by the engagement of checkpoint receptors, such as PD-1, or inhibitory HLA-specific receptors, as the interaction with NK cells could even induce tumor cells to increase the expression of HLA class I molecules, including HLA-G and HLA-E [216]. The combined use of mAbs targeting tumor antigens (e.g., EGFR) and NKG2A have been proposed as a novel approach to increase both NK cell-mediated ADCC and the T cell responses of NKG2A+ T cells [217]. A further increase of CD16-mediated NK cell activation may be obtained by using multifunctional NK cell engagers that have been shown to enhance ADCC efficiency. Thus, a trifunctional engager (targeting CD16, NKp46 and a tumor Ag) has been shown to drive NK cell-mediated anti-tumor activity in mouse models of both invasive and solid tumors [218]. A trifunctional molecule combining IL-15 with CD16 and CD33 engagers has also been constructed to support NK cell response in myelodysplastic syndrome (MDS) [219]. This molecule, besides stimulating NK cell effector function, also promotes NK cell proliferation, but, remarkably, without inducing the IL-15-related upregulation of the checkpoint receptor TIGIT. This was an important issue, as TIGIT suppresses NK cells via interaction with MDSC in MDS.

MDSCs have also been demonstrated to interfere with the anti-leukemic activity of NK cells in αβT cell and B cell HLA-haploidentical HSCT [220]. This type of HSCT is used to treat leukemic patients lacking an HLA-compatible donor. Mobilized HSCs are depleted of B and αβT cells, but contain the donor’s NK cells and γδT cells that may support anti-leukemia activity in the early stages of transplantation. Remarkably, a significant proportion of polymorphonuclear (PMN)-MDSC cells may be observed among the myeloid cells present in these HSC grafts. These PMN-MDSC cells appear to suppress mature NK cells, but, seemingly, had limited effects on the maturation of NK cells from HSCs. Other regulatory cells may play a crucial role in the resistance to NK cell therapies. For instance, Tregs, which can undergo IL-2-driven expansion in vivo [221,222], may be responsible for the frustrating results obtained in clinical studies combining rituximab with low-intermediate doses of IL-2 to promote NK cell expansion and increased ADCC [221]. Similarly, cetuximab treatment increased the frequency of CTLA-4+ Tregs that suppressed cetuximab-induced ADCC and correlated with poor clinical outcomes [223].

Checkpoint inhibitors may rescue NK cells from exhaustion, unleashing their response and their ADCC activity [188,190,224]. However, it should be considered that blocking one type of receptor–ligand pair may not be always sufficient due to the presence of multiple receptor–ligand pairs within the tumor with variable tissue and immune cell distributions. This is suggested by a multiplexed expression analysis of PD-1, TIM3 and LAG-3 in NSCLC, indicating a negative association between high LAG-3 expression and sensitivity to PD-1 axis blockade [225]. PD-L1 expression has also recently been shown to be induced on NK cells, providing a possible explanation for the clinical response to PD-1 inhibition in patients bearing PD-L1-negative tumor [226]. Checkpoint blockade-based therapy has been observed to increase the tumor regression induced by radiotherapy [214]. For decades, the so-called abscopal effect was observed in a low percentage of patients treated with radiotherapy. This phenomenon refers to the regression of tumors outside of the focus of radiotherapy treatment due to, at least in part, the exposure and recognition of neoantigens by the immune system. The combination of checkpoint blockade and radiotherapy has shown encouraging results in patients with hematological malignancies and solid tumors, such as metastatic melanoma and breast cancer [227,228,229,230].

## 5. Conclusions

Immunotherapy has been a major breakthrough in cancer. Owing to their rapid and potent cytotoxic activity, NK cells may be efficient targets for cancer immunotherapy. The expansion, persistence and capability of NK cells to infiltrate solid tumors in vivo seem to be key factors influencing the clinical response. Thus, different therapeutic strategies and procedures are being employed to boost the anti-tumor activity of NK cells. Despite some encouraging clinical results, especially in hematologic malignancies, a high degree of resistance to NK cell-based therapies has often been reported. These mechanisms of resistance may significantly vary according to therapy and disease, but there are common determinants affecting the efficacy of/resistance to NK cell therapy. The genetic profile, which mainly determines the functional activity of NK cells; the biology of the cancer cells; the tumor microenvironment; and their complex interactions with the immune system are common factors influencing resistance to NK cell therapy. This clearly means that therapy efficacy and resistance are specific to a particular patient and disease, suggesting that the selection of suitable patients is crucial for the success of immunotherapy. Furthermore, the concept that multiple concomitant escape mechanisms may contribute to resistance is driving studies towards the design of complex therapeutic strategies to avoid immunosuppression, to increase tumor cell susceptibility to NK cell attack, and to produce long-lasting effective NK cell effectors [52,59,231,232]. In this view, multiple therapeutic tools—including cytokines or cytokine-based agonists, anti-cancer drugs, multifunctional NK cell engagers, checkpoint inhibitors, CAR-NK cells, memory-like NK cells and allo-reactive NK cells—need to be properly coordinated and adapted to the different pathological conditions. Thus, a major effort needs to be made in the future to identify the mechanisms of resistance in different contexts in order to devise effective therapeutic combinations, optimize schedules and procedures, and improve efficacy.

## Figures and Tables

**Table 1:** Resistance to natural killer (NK) cell-based therapy.

Mechanisms	Therapy	Main Resistance Mechanisms	Overcoming Resistance
ADCC	MAbs	- Low NK cell numbers or activity - *FCGR3A* polymorphisms - Shedding or low expression of CD16 - Loss or modulation of expression of the target antigen - Expression of anti-apoptotic proteins by cancer cells - Tumor microenvironment interactions - Immunosuppressive cytokines and microenvironment - Expression of checkpoint proteins and inhibitory receptors	- Improving mAbs with increased affinity - Combination with immunomodulatory drugs and cytokines - Using multitarget BiKEs and TRiKEs - Metalloproteinase inhibitors to avoid CD16 shedding - Using pro-apoptotic drugs - Targeting inhibitory and immunosuppressive proteins
Missing-self recognition	HSTC allogenic NK cell transfer	- KIR/HLA repertoire - Presence of specific inhibitory KIR genes - Increased expression of non-classical MHC class I molecules - Short lifespan (adoptive transfer) - Poor persistence and trafficking (adoptive transfer)	- Activation and expansion of NK cells - Using allogenic HSTC - Using anti-KIR antibodies
Activating receptors	NK cell transfer Immuno-modulatory drugs and cytokines	- Downregulation of activating receptors or ligands - Increased expression of checkpoint proteins and inhibitory proteins - Expression of anti-apoptotic proteins by cancer cells - Tumor microenvironment interactions - Immunosuppressive cytokines and microenvironment - Short lifespan (adoptive transfer) - Poor persistence and trafficking (adoptive transfer)	- Activation and expansion of NK cells - Induction of NKG2D ligand expression - Using pro-apoptotic drugs - Targeting inhibitory and immunosuppressive proteins - Using NK cell lines (i.e., NK-92) - Therapy with NKG2D CAR-NK cells
Chimeric antigenic receptors	CAR-NK cells	- Short lifespan - Poor activation, persistence and trafficking	- IL-15-expressing CAR-NK cells - Using NK-92 cells as carriers - Combination with mAbs

## References

[1] Cantoni C., Grauwet K., Pietra G., Parodi M., Mingari M.C., Maria A.D., Favoreel H., Vitale M. (2015). Role of NK cells in immunotherapy and virotherapy of solid tumors. Immunotherapy.

[2] Chiossone L., Dumas P.Y., Vienne M., Vivier E. (2018). Natural killer cells and other innate lymphoid cells in cancer. Nat. Rev. Immunol..

[3] Karre K., Ljunggren H.G., Piontek G., Kiessling R. (1986). Selective rejection of H-2-deficient lymphoma variants suggests alternative immune defence strategy. Nature.

[4] Sun J.C., Lanier L.L. (2011). NK cell development, homeostasis and function: Parallels with CD8(+) T cells. Nat. Rev. Immunol..

[5] Moretta L., Locatelli F., Pende D., Sivori S., Falco M., Bottino C., Mingari M.C., Moretta A. (2011). Human NK receptors: From the molecules to the therapy of high risk leukemias. FEBS Lett..

[6] Sivori S., Vacca P., Del Zotto G., Munari E., Mingari M.C., Moretta L. (2019). Human NK cells: Surface receptors, inhibitory checkpoints, and translational applications. Cell. Mol. Immunol..

[7] Muntasell A., Ochoa M.C., Cordeiro L., Berraondo P., Lopez-Diaz de Cerio A., Cabo M., Lopez-Botet M., Melero I. (2017). Targeting NK-cell checkpoints for cancer immunotherapy. Curr. Opin. Immunol..

[8] Hsu J., Hodgins J.J., Marathe M., Nicolai C.J., Bourgeois-Daigneault M.C., Trevino T.N., Azimi C.S., Scheer A.K., Randolph H.E., Thompson T.W. (2018). Contribution of NK cells to immunotherapy mediated by PD-1/PD-L1 blockade. J. Clin. Investig..

[9] Quatrini L., Wieduwild E., Escaliere B., Filtjens J., Chasson L., Laprie C., Vivier E., Ugolini S. (2018). Endogenous glucocorticoids control host resistance to viral infection through the tissue-specific regulation of PD-1 expression on NK cells. Nat. Immunol..

[10] Huergo-Zapico L., Acebes-Huerta A., Lopez-Soto A., Villa-Alvarez M., Gonzalez-Rodriguez A.P., Gonzalez S. (2014). Molecular Bases for the Regulation of NKG2D Ligands in Cancer. Front. Immunol..

[11] Lopez-Soto A., Huergo-Zapico L., Acebes-Huerta A., Villa-Alvarez M., Gonzalez S. (2015). NKG2D signaling in cancer immunosurveillance. Int. J. Cancer.

[12] Moretta A., Bottino C., Vitale M., Pende D., Cantoni C., Mingari M.C., Biassoni R., Moretta L. (2001). Activating receptors and coreceptors involved in human natural killer cell-mediated cytolysis. Annu. Rev. Immunol..

[13] Gasser S., Orsulic S., Brown E.J., Raulet D.H. (2005). The DNA damage pathway regulates innate immune system ligands of the NKG2D receptor. Nature.

[14] Zingoni A., Cecere F., Vulpis E., Fionda C., Molfetta R., Soriani A., Petrucci M.T., Ricciardi M.R., Fuerst D., Amendola M.G. (2015). Genotoxic Stress Induces Senescence-Associated ADAM10-Dependent Release of NKG2D MIC Ligands in Multiple Myeloma Cells. J. Immunol..

[15] Laurent S., Queirolo P., Boero S., Salvi S., Piccioli P., Boccardo S., Minghelli S., Morabito A., Fontana V., Pietra G. (2013). The engagement of CTLA-4 on primary melanoma cell lines induces antibody-dependent cellular cytotoxicity and TNF-alpha production. J. Transl. Med..

[16] Romano E., Kusio-Kobialka M., Foukas P.G., Baumgaertner P., Meyer C., Ballabeni P., Michielin O., Weide B., Romero P., Speiser D.E. (2015). Ipilimumab-dependent cell-mediated cytotoxicity of regulatory T cells ex vivo by nonclassical monocytes in melanoma patients. Proc. Natl. Acad. Sci. USA.

[17] Lorenzo-Herrero S., Lopez-Soto A., Sordo-Bahamonde C., Gonzalez-Rodriguez A.P., Vitale M., Gonzalez S. (2018). NK Cell-Based Immunotherapy in Cancer Metastasis. Cancers.

[18] Gonzalez-Rodriguez A.P., Villa-Alvarez M., Sordo-Bahamonde C., Lorenzo-Herrero S., Gonzalez S. (2019). NK Cells in the Treatment of Hematological Malignancies. J. Clin. Med..

[19] Lo Nigro C., Macagno M., Sangiolo D., Bertolaccini L., Aglietta M., Merlano M.C. (2019). NK-mediated antibody-dependent cell-mediated cytotoxicity in solid tumors: Biological evidence and clinical perspectives. Ann. Transl. Med..

[20] Nayyar G., Chu Y., Cairo M.S. (2019). Overcoming Resistance to Natural Killer Cell Based Immunotherapies for Solid Tumors. Front. Oncol..

[21] Giles A.J., Hao S., Padget M., Song H., Zhang W., Lynes J., Sanchez V., Liu Y., Jung J., Cao X. (2019). Efficient ADCC killing of meningioma by avelumab and a high-affinity natural killer cell line, haNK. JCI Insight.

[22] Weiner L.M., Surana R., Wang S. (2010). Monoclonal antibodies: Versatile platforms for cancer immunotherapy. Nat. Rev. Immunol..

[23] Pierpont T.M., Limper C.B., Richards K.L. (2018). Past, Present, and Future of Rituximab-The World’s First Oncology Monoclonal Antibody Therapy. Front. Oncol..

[24] Weiner G.J. (2010). Rituximab: Mechanism of action. Semin. Hematol..

[25] Koch J., Tesar M. (2017). Recombinant Antibodies to Arm Cytotoxic Lymphocytes in Cancer Immunotherapy. Transfus. Med. Hemotherapy.

[26] Gleason M.K., Verneris M.R., Todhunter D.A., Zhang B., McCullar V., Zhou S.X., Panoskaltsis-Mortari A., Weiner L.M., Vallera D.A., Miller J.S. (2012). Bispecific and trispecific killer cell engagers directly activate human NK cells through CD16 signaling and induce cytotoxicity and cytokine production. Mol. Cancer Ther..

[27] Chan W.K., Kang S., Youssef Y., Glankler E.N., Barrett E.R., Carter A.M., Ahmed E.H., Prasad A., Chen L., Zhang J. (2018). A CS1-NKG2D Bispecific Antibody Collectively Activates Cytolytic Immune Cells against Multiple Myeloma. Cancer Immunol. Res..

[28] Bottcher J.P., Bonavita E., Chakravarty P., Blees H., Cabeza-Cabrerizo M., Sammicheli S., Rogers N.C., Sahai E., Zelenay S., Reis e Sousa C. (2018). NK Cells Stimulate Recruitment of cDC1 into the Tumor Microenvironment Promoting Cancer Immune Control. Cell.

[29] Benson D.M., Bakan C.E., Mishra A., Hofmeister C.C., Efebera Y., Becknell B., Baiocchi R.A., Zhang J., Yu J., Smith M.K. (2010). The PD-1/PD-L1 axis modulates the natural killer cell versus multiple myeloma effect: A therapeutic target for CT-011, a novel monoclonal anti-PD-1 antibody. Blood.

[30] Liu Y., Cheng Y., Xu Y., Wang Z., Du X., Li C., Peng J., Gao L., Liang X., Ma C. (2017). Increased expression of programmed cell death protein 1 on NK cells inhibits NK-cell-mediated anti-tumor function and indicates poor prognosis in digestive cancers. Oncogene.

[31] Barry K.C., Hsu J., Broz M.L., Cueto F.J., Binnewies M., Combes A.J., Nelson A.E., Loo K., Kumar R., Rosenblum M.D. (2018). A natural killer-dendritic cell axis defines checkpoint therapy-responsive tumor microenvironments. Nat. Med..

[32] Romagne F., Andre P., Spee P., Zahn S., Anfossi N., Gauthier L., Capanni M., Ruggeri L., Benson D.M., Blaser B.W. (2009). Preclinical characterization of 1-7F9, a novel human anti-KIR receptor therapeutic antibody that augments natural killer-mediated killing of tumor cells. Blood.

[33] McWilliams E.M., Mele J.M., Cheney C., Timmerman E.A., Fiazuddin F., Strattan E.J., Mo X., Byrd J.C., Muthusamy N., Awan F.T. (2016). Therapeutic CD94/NKG2A blockade improves natural killer cell dysfunction in chronic lymphocytic leukemia. Oncoimmunology.

[34] Weiden P.L., Flournoy N., Thomas E.D., Prentice R., Fefer A., Buckner C.D., Storb R. (1979). Antileukemic effect of graft-versus-host disease in human recipients of allogeneic-marrow grafts. N. Engl. J. Med..

[35] Ruggeri L., Mancusi A., Burchielli E., Capanni M., Carotti A., Aloisi T., Aversa F., Martelli M.F., Velardi A. (2008). NK cell alloreactivity and allogeneic hematopoietic stem cell transplantation. Blood Cells Mol. Dis..

[36] Kloess S., Kretschmer A., Stahl L., Fricke S., Koehl U. (2019). CAR-Expressing Natural Killer Cells for Cancer Retargeting. Transfus. Med. Hemotherapy.

[37] Jiang T., Zhou C., Ren S. (2016). Role of IL-2 in cancer immunotherapy. Oncoimmunology.

[38] Romee R., Leong J.W., Fehniger T.A. (2014). Utilizing cytokines to function-enable human NK cells for the immunotherapy of cancer. Scientifica.

[39] Parihar R., Dierksheide J., Hu Y., Carson W.E. (2002). IL-12 enhances the natural killer cell cytokine response to Ab-coated tumor cells. J. Clin. Investig..

[40] Roda J.M., Parihar R., Lehman A., Mani A., Tridandapani S., Carson W.E. (2006). Interleukin-21 enhances NK cell activation in response to antibody-coated targets. J. Immunol..

[41] Robinson T.O., Schluns K.S. (2017). The potential and promise of IL-15 in immuno-oncogenic therapies. Immunol. Lett..

[42] Margolin K., Morishima C., Velcheti V., Miller J.S., Lee S.M., Silk A.W., Holtan S.G., Lacroix A.M., Fling S.P., Kaiser J.C. (2018). Phase I Trial of ALT-803, A Novel Recombinant IL15 Complex, in Patients with Advanced Solid Tumors. Clin. Cancer Res..

[43] Pinette A., McMichael E., Courtney N.B., Duggan M., Benner B.N., Choueiry F., Yu L., Abood D., Mace T.A., Carson W.E. (2019). An IL-15-based superagonist ALT-803 enhances the NK cell response to cetuximab-treated squamous cell carcinoma of the head and neck. Cancer Immunol. Immunother..

[44] Hagner P.R., Chiu H., Ortiz M., Apollonio B., Wang M., Couto S., Waldman M.F., Flynt E., Ramsay A.G., Trotter M. (2017). Activity of lenalidomide in mantle cell lymphoma can be explained by NK cell-mediated cytotoxicity. Br. J. Haematol..

[45] Gribben J.G., Fowler N., Morschhauser F. (2015). Mechanisms of Action of Lenalidomide in B-Cell Non-Hodgkin Lymphoma. J. Clin. Oncol..

[46] Acebes-Huerta A., Huergo-Zapico L., Gonzalez-Rodriguez A.P., Fernandez-Guizan A., Payer A.R., Lopez-Soto A., Gonzalez S. (2014). Lenalidomide induces immunomodulation in chronic lymphocytic leukemia and enhances antitumor immune responses mediated by NK and CD4 T cells. Biomed Res. Int..

[47] Gonzalez-Rodriguez A.P., Payer A.R., Acebes-Huerta A., Huergo-Zapico L., Villa-Alvarez M., Gonzalez-Garcia E., Gonzalez S. (2013). Lenalidomide and chronic lymphocytic leukemia. Biomed Res. Int..

[48] Giuliani M., Janji B., Berchem G. (2017). Activation of NK cells and disruption of PD-L1/PD-1 axis: Two different ways for lenalidomide to block myeloma progression. Oncotarget.

[49] Villa-Alvarez M., Sordo-Bahamonde C., Lorenzo-Herrero S., Gonzalez-Rodriguez A.P., Payer A.R., Gonzalez-Garcia E., Villa-Alvarez M.C., Lopez-Soto A., Gonzalez S. (2018). Ig-Like Transcript 2 (ILT2) Blockade and Lenalidomide Restore NK Cell Function in Chronic Lymphocytic Leukemia. Front. Immunol..

[50] Chiu H., Trisal P., Bjorklund C., Carrancio S., Torano E.G., Guarinos C., Papazoglou D., Hagner P.R., Beldi-Ferchiou A., Tarte K. (2019). Combination lenalidomide-rituximab immunotherapy activates anti-tumour immunity and induces tumour cell death by complementary mechanisms of action in follicular lymphoma. Br. J. Haematol..

[51] Fionda C., Abruzzese M.P., Zingoni A., Cecere F., Vulpis E., Peruzzi G., Soriani A., Molfetta R., Paolini R., Ricciardi M.R. (2015). The IMiDs targets IKZF-1/3 and IRF4 as novel negative regulators of NK cell-activating ligands expression in multiple myeloma. Oncotarget.

[52] Cifaldi L., Locatelli F., Marasco E., Moretta L., Pistoia V. (2017). Boosting Natural Killer Cell-Based Immunotherapy with Anticancer Drugs: A Perspective. Trends Mol. Med..

[53] Klingemann H. (2014). Are natural killer cells superior CAR drivers?. Oncoimmunology.

[54] Ruggeri L., Capanni M., Casucci M., Volpi I., Tosti A., Perruccio K., Urbani E., Negrin R.S., Martelli M.F., Velardi A. (1999). Role of natural killer cell alloreactivity in HLA-mismatched hematopoietic stem cell transplantation. Blood.

[55] Ruggeri L., Capanni M., Urbani E., Perruccio K., Shlomchik W.D., Tosti A., Posati S., Rogaia D., Frassoni F., Aversa F. (2002). Effectiveness of donor natural killer cell alloreactivity in mismatched hematopoietic transplants. Science.

[56] Rezvani K., Rouce R.H. (2015). The Application of Natural Killer Cell Immunotherapy for the Treatment of Cancer. Front. Immunol..

[57] Stojanovic A., Cerwenka A. (2011). Natural killer cells and solid tumors. J. Innate Immun..

[58] Becker P.S., Suck G., Nowakowska P., Ullrich E., Seifried E., Bader P., Tonn T., Seidl C. (2016). Selection and expansion of natural killer cells for NK cell-based immunotherapy. Cancer Immunol. Immunother..

[59] Romee R., Rosario M., Berrien-Elliott M.M., Wagner J.A., Jewell B.A., Schappe T., Leong J.W., Abdel-Latif S., Schneider S.E., Willey S. (2016). Cytokine-induced memory-like natural killer cells exhibit enhanced responses against myeloid leukemia. Sci. Transl. Med..

[60] Schmidt-Wolf I.G., Negrin R.S., Kiem H.P., Blume K.G., Weissman I.L. (1991). Use of a SCID mouse/human lymphoma model to evaluate cytokine-induced killer cells with potent antitumor cell activity. J. Exp. Med..

[61] Uherek C., Tonn T., Uherek B., Becker S., Schnierle B., Klingemann H.G., Wels W. (2002). Retargeting of natural killer-cell cytolytic activity to ErbB2-expressing cancer cells results in efficient and selective tumor cell destruction. Blood.

[62] Maki G., Klingemann H.G., Martinson J.A., Tam Y.K. (2001). Factors regulating the cytotoxic activity of the human natural killer cell line, NK-92. J. Hematotherapy Stem Cell Res..

[63] Cooley S., Xiao F., Pitt M., Gleason M., McCullar V., Bergemann T.L., McQueen K.L., Guethlein L.A., Parham P., Miller J.S. (2007). A subpopulation of human peripheral blood NK cells that lacks inhibitory receptors for self-MHC is developmentally immature. Blood.

[64] Suck G., Odendahl M., Nowakowska P., Seidl C., Wels W.S., Klingemann H.G., Tonn T. (2016). NK-92: An ’off-the-shelf therapeutic’ for adoptive natural killer cell-based cancer immunotherapy. Cancer Immunol. Immunother..

[65] Boissel L., Betancur-Boissel M., Lu W., Krause D.S., Van Etten R.A., Wels W.S., Klingemann H. (2013). Retargeting NK-92 cells by means of CD19- and CD20-specific chimeric antigen receptors compares favorably with antibody-dependent cellular cytotoxicity. Oncoimmunology.

[66] Boissel L., Betancur M., Wels W.S., Tuncer H., Klingemann H. (2009). Transfection with mRNA for CD19 specific chimeric antigen receptor restores NK cell mediated killing of CLL cells. Leuk. Res..

[67] Rezvani A.R., Maloney D.G. (2011). Rituximab resistance. Best Pract. Res. Clin. Haematol..

[68] Arribas J., Baselga J., Pedersen K., Parra-Palau J.L. (2011). p95HER2 and breast cancer. Cancer Res..

[69] Nakadate Y., Kodera Y., Kitamura Y., Shirasawa S., Tachibana T., Tamura T., Koizumi F. (2014). KRAS mutation confers resistance to antibody-dependent cellular cytotoxicity of cetuximab against human colorectal cancer cells. Int. J. Cancer.

[70] Braig F., Kriegs M., Voigtlaender M., Habel B., Grob T., Biskup K., Blanchard V., Sack M., Thalhammer A., Ben Batalla I. (2017). Cetuximab Resistance in Head and Neck Cancer Is Mediated by EGFR-K521 Polymorphism. Cancer Res..

[71] Roederer M., Quaye L., Mangino M., Beddall M.H., Mahnke Y., Chattopadhyay P., Tosi I., Napolitano L., Terranova Barberio M., Menni C. (2015). The genetic architecture of the human immune system: A bioresource for autoimmunity and disease pathogenesis. Cell.

[72] Moussa P., Marton J., Vidal S.M., Fodil-Cornu N. (2012). Genetic dissection of NK cell responses. Front. Immunol..

[73] Ferreira M.A., Mangino M., Brumme C.J., Zhao Z.Z., Medland S.E., Wright M.J., Nyholt D.R., Gordon S., Campbell M., McEvoy B.P. (2010). Quantitative trait loci for CD4:CD8 lymphocyte ratio are associated with risk of type 1 diabetes and HIV-1 immune control. Am. J. Hum. Genet..

[74] Hall M.A., Ahmadi K.R., Norman P., Snieder H., MacGregor A.J., Vaughan R.W., Spector T.D., Lanchbury J.S. (2000). Genetic influence on peripheral blood T lymphocyte levels. Genes Immun..

[75] Xia Z., Liu Q., Berger C.T., Keenan B.T., Kaliszewska A., Cheney P.C., Srivastava G.P., Castillo I.W., De Jager P.L., Alter G. (2012). A 17q12 allele is associated with altered NK cell subsets and function. J. Immunol..

[76] Imai K., Matsuyama S., Miyake S., Suga K., Nakachi K. (2000). Natural cytotoxic activity of peripheral-blood lymphocytes and cancer incidence: An 11-year follow-up study of a general population. Lancet.

[77] Klanova M., Oestergaard M.Z., Trneny M., Hiddemann W., Marcus R., Sehn L.H., Vitolo U., Bazeos A., Goede V., Zeuner H. (2019). Prognostic Impact of Natural Killer Cell Count in Follicular Lymphoma and Diffuse Large B-cell Lymphoma Patients Treated with Immunochemotherapy. Clin. Cancer Res..

[78] Coca S., Perez-Piqueras J., Martinez D., Colmenarejo A., Saez M.A., Vallejo C., Martos J.A., Moreno M. (1997). The prognostic significance of intratumoral natural killer cells in patients with colorectal carcinoma. Cancer.

[79] Salgado R., Denkert C., Campbell C., Savas P., Nuciforo P., Aura C., de Azambuja E., Eidtmann H., Ellis C.E., Baselga J. (2015). Tumor-Infiltrating Lymphocytes and Associations With Pathological Complete Response and Event-Free Survival in HER2-Positive Early-Stage Breast Cancer Treated With Lapatinib and Trastuzumab: A Secondary Analysis of the NeoALTTO Trial. JAMA Oncol..

[80] Denkert C., von Minckwitz G., Darb-Esfahani S., Lederer B., Heppner B.I., Weber K.E., Budczies J., Huober J., Klauschen F., Furlanetto J. (2018). Tumour-infiltrating lymphocytes and prognosis in different subtypes of breast cancer: A pooled analysis of 3771 patients treated with neoadjuvant therapy. Lancet Oncol..

[81] Danielou-Lazareth A., Henry G., Geromin D., Khaznadar Z., Briere J., Tamouza R., Cayuela J.M., Thieblemont C., Toubert A., Dulphy N. (2013). At diagnosis, diffuse large B-cell lymphoma patients show impaired rituximab-mediated NK-cell cytotoxicity. Eur. J. Immunol..

[82] Hatjiharissi E., Xu L., Santos D.D., Hunter Z.R., Ciccarelli B.T., Verselis S., Modica M., Cao Y., Manning R.J., Leleu X. (2007). Increased natural killer cell expression of CD16, augmented binding and ADCC activity to rituximab among individuals expressing the Fc{gamma}RIIIa-158 V/V and V/F polymorphism. Blood.

[83] Cartron G., Dacheux L., Salles G., Solal-Celigny P., Bardos P., Colombat P., Watier H. (2002). Therapeutic activity of humanized anti-CD20 monoclonal antibody and polymorphism in IgG Fc receptor FcgammaRIIIa gene. Blood.

[84] Weng W.K., Levy R. (2003). Two immunoglobulin G fragment C receptor polymorphisms independently predict response to rituximab in patients with follicular lymphoma. J. Clin. Oncol..

[85] Treon S.P., Hansen M., Branagan A.R., Verselis S., Emmanouilides C., Kimby E., Frankel S.R., Touroutoglou N., Turnbull B., Anderson K.C. (2005). Polymorphisms in FcgammaRIIIA (CD16) receptor expression are associated with clinical response to rituximab in Waldenstrom’s macroglobulinemia. J. Clin. Oncol..

[86] Kim D.H., Jung H.D., Kim J.G., Lee J.J., Yang D.H., Park Y.H., Do Y.R., Shin H.J., Kim M.K., Hyun M.S. (2006). FCGR3A gene polymorphisms may correlate with response to frontline R-CHOP therapy for diffuse large B-cell lymphoma. Blood.

[87] Farag S.S., Flinn I.W., Modali R., Lehman T.A., Young D., Byrd J.C. (2004). Fc gamma RIIIa and Fc gamma RIIa polymorphisms do not predict response to rituximab in B-cell chronic lymphocytic leukemia. Blood.

[88] Oboshi W., Watanabe T., Yukimasa N., Ueno I., Aki K., Tada T., Hosoi E. (2016). SNPs rs4656317 and rs12071048 located within an enhancer in FCGR3A are in strong linkage disequilibrium with rs396991 and influence NK cell-mediated ADCC by transcriptional regulation. Hum. Immunol..

[89] Musolino A., Naldi N., Bortesi B., Pezzuolo D., Capelletti M., Missale G., Laccabue D., Zerbini A., Camisa R., Bisagni G. (2008). Immunoglobulin G fragment C receptor polymorphisms and clinical efficacy of trastuzumab-based therapy in patients with HER-2/neu-positive metastatic breast cancer. J. Clin. Oncol..

[90] Tamura K., Shimizu C., Hojo T., Akashi-Tanaka S., Kinoshita T., Yonemori K., Kouno T., Katsumata N., Ando M., Aogi K. (2010). FcγR2A and 3A polymorphisms predict clinical outcome of trastuzumab in both neoadjuvant and metastatic settings in patients with HER2-positive breast cancer. Ann. Oncol..

[91] Bibeau F., Lopez-Crapez E., Di Fiore F., Thezenas S., Ychou M., Blanchard F., Lamy A., Penault-Llorca F., Frebourg T., Michel P. (2009). Impact of Fc{gamma}RIIa-Fc{gamma}RIIIa polymorphisms and KRAS mutations on the clinical outcome of patients with metastatic colorectal cancer treated with cetuximab plus irinotecan. J. Clin. Oncol..

[92] Lopez-Albaitero A., Lee S.C., Morgan S., Grandis J.R., Gooding W.E., Ferrone S., Ferris R.L. (2009). Role of polymorphic Fc gamma receptor IIIa and EGFR expression level in cetuximab mediated, NK cell dependent in vitro cytotoxicity of head and neck squamous cell carcinoma cells. Cancer Immunol. Immunother..

[93] Umaña P., Jean–Mairet J., Moudry R., Amstutz H., Bailey J.E. (1999). Engineered glycoforms of an antineuroblastoma IgG1 with optimized antibody-dependent cellular cytotoxic activity. Nat. Biotechnol..

[94] Prica A., Crump M. (2019). Improving CD20 antibody therapy: Obinutuzumab in lymphoproliferative disorders. Leuk. Lymphoma.

[95] Anfossi N., Andre P., Guia S., Falk C.S., Roetynck S., Stewart C.A., Breso V., Frassati C., Reviron D., Middleton D. (2006). Human NK cell education by inhibitory receptors for MHC class I. Immunity.

[96] Forlenza C.J., Boudreau J.E., Zheng J., Le Luduec J.B., Chamberlain E., Heller G., Cheung N.K., Hsu K.C. (2016). KIR3DL1 Allelic Polymorphism and HLA-B Epitopes Modulate Response to Anti-GD2 Monoclonal Antibody in Patients With Neuroblastoma. J. Clin. Oncol..

[97] Levinson R.D., Yung M., Meguro A., Ashouri E., Yu F., Mizuki N., Ohno S., Rajalingam R. (2016). KIR and HLA Genotypes Implicated in Reduced Killer Lymphocytes Immunity Are Associated with Vogt-Koyanagi-Harada Disease. PLoS ONE.

[98] Erbe A.K., Wang W., Reville P.K., Carmichael L., Kim K., Mendonca E.A., Song Y., Hank J.A., London W.B., Naranjo A. (2017). HLA-Bw4-I-80 Isoform Differentially Influences Clinical Outcome As Compared to HLA-Bw4-T-80 and HLA-A-Bw4 Isoforms in Rituximab or Dinutuximab-Based Cancer Immunotherapy. Front. Immunol..

[99] Du J., Lopez-Verges S., Pitcher B.N., Johnson J., Jung S.H., Zhou L., Hsu K., Czuczman M.S., Cheson B., Kaplan L. (2014). CALGB 150905 (Alliance): Rituximab broadens the antilymphoma response by activating unlicensed NK cells. Cancer Immunol. Res..

[100] Terszowski G., Klein C., Stern M. (2014). KIR/HLA interactions negatively affect rituximab- but not GA101 (obinutuzumab)-induced antibody-dependent cellular cytotoxicity. J. Immunol..

[101] Brunstein C.G., Wagner J.E., Weisdorf D.J., Cooley S., Noreen H., Barker J.N., DeFor T., Verneris M.R., Blazar B.R., Miller J.S. (2009). Negative effect of KIR alloreactivity in recipients of umbilical cord blood transplant depends on transplantation conditioning intensity. Blood.

[102] Huang X.J., Zhao X.Y., Liu D.H., Liu K.Y., Xu L.P. (2007). Deleterious effects of KIR ligand incompatibility on clinical outcomes in haploidentical hematopoietic stem cell transplantation without in vitro T-cell depletion. Leukemia.

[103] Faridi R.M., Kemp T.J., Dharmani-Khan P., Lewis V., Tripathi G., Rajalingam R., Daly A., Berka N., Storek J., Masood Khan F. (2016). Donor-Recipient Matching for KIR Genotypes Reduces Chronic GVHD and Missing Inhibitory KIR Ligands Protect against Relapse after Myeloablative, HLA Matched Hematopoietic Cell Transplantation. PLoS ONE.

[104] Cooley S., Trachtenberg E., Bergemann T.L., Saeteurn K., Klein J., Le C.T., Marsh S.G., Guethlein L.A., Parham P., Miller J.S. (2009). Donors with group B KIR haplotypes improve relapse-free survival after unrelated hematopoietic cell transplantation for acute myelogenous leukemia. Blood.

[105] Venstrom J.M., Pittari G., Gooley T.A., Chewning J.H., Spellman S., Haagenson M., Gallagher M.M., Malkki M., Petersdorf E., Dupont B. (2012). HLA-C-dependent prevention of leukemia relapse by donor activating KIR2DS1. New Engl. J. Med..

[106] Hanahan D., Weinberg R.A. (2000). The hallmarks of cancer. Cell.

[107] Zhao W.L., Daneshpouy M.E., Mounier N., Briere J., Leboeuf C., Plassa L.F., Turpin E., Cayuela J.M., Ameisen J.C., Gisselbrecht C. (2004). Prognostic significance of bcl-xL gene expression and apoptotic cell counts in follicular lymphoma. Blood.

[108] Gisselbrecht C., Glass B., Mounier N., Singh Gill D., Linch D.C., Trneny M., Bosly A., Ketterer N., Shpilberg O., Hagberg H. (2010). Salvage regimens with autologous transplantation for relapsed large B-cell lymphoma in the rituximab era. J. Clin. Oncol..

[109] Bannerji R., Kitada S., Flinn I.W., Pearson M., Young D., Reed J.C., Byrd J.C. (2003). Apoptotic-regulatory and complement-protecting protein expression in chronic lymphocytic leukemia: Relationship to in vivo rituximab resistance. J. Clin. Oncol..

[110] Berns K., Horlings H.M., Hennessy B.T., Madiredjo M., Hijmans E.M., Beelen K., Linn S.C., Gonzalez-Angulo A.M., Stemke-Hale K., Hauptmann M. (2007). A functional genetic approach identifies the PI3K pathway as a major determinant of trastuzumab resistance in breast cancer. Cancer Cell.

[111] Brand T.M., Lida M., Wheeler D.L. (2011). Molecular mechanisms of resistance to the EGFR monoclonal antibody cetuximab. Cancer Biol. Ther..

[112] Jazirehi A.R., Vega M.I., Bonavida B. (2007). Development of rituximab-resistant lymphoma clones with altered cell signaling and cross-resistance to chemotherapy. Cancer Res..

[113] Karapetis C.S., Khambata-Ford S., Jonker D.J., O’Callaghan C.J., Tu D., Tebbutt N.C., Simes R.J., Chalchal H., Shapiro J.D., Robitaille S. (2008). K-ras mutations and benefit from cetuximab in advanced colorectal cancer. N. Engl. J. Med..

[114] Luque-Cabal M., Garcia-Teijido P., Fernandez-Perez Y., Sanchez-Lorenzo L., Palacio-Vazquez I. (2016). Mechanisms Behind the Resistance to Trastuzumab in HER2-Amplified Breast Cancer and Strategies to Overcome It. Clin. Med. Insights Oncol..

[115] Olejniczak S.H., Hernandez-Ilizaliturri F.J., Clements J.L., Czuczman M.S. (2008). Acquired resistance to rituximab is associated with chemotherapy resistance resulting from decreased Bax and Bak expression. Clin. Cancer Res..

[116] Vega M.I., Jazirehi A.R., Huerta-Yepez S., Bonavida B. (2005). Rituximab-induced inhibition of YY1 and Bcl-xL expression in Ramos non-Hodgkin’s lymphoma cell line via inhibition of NF-kappa B activity: Role of YY1 and Bcl-xL in Fas resistance and chemoresistance, respectively. J. Immunol..

[117] Obexer P., Ausserlechner M.J. (2014). X-linked inhibitor of apoptosis protein - a critical death resistance regulator and therapeutic target for personalized cancer therapy. Front. Oncol..

[118] Evans M.K., Sauer S.J., Nath S., Robinson T.J., Morse M.A., Devi G.R. (2016). X-linked inhibitor of apoptosis protein mediates tumor cell resistance to antibody-dependent cellular cytotoxicity. Cell Death Dis..

[119] Li R., Ruttinger D., Urba W., Fox B.A., Hu H.M. (2004). Targeting and amplification of immune killing of tumor cells by pro-Smac. Int. J. Cancer.

[120] Pan W., Luo Q., Yan X., Yuan L., Yi H., Zhang L., Li B., Zhang Y., Sun J., Qiu M.Z. (2018). A novel SMAC mimetic APG-1387 exhibits dual antitumor effect on HBV-positive hepatocellular carcinoma with high expression of cIAP2 by inducing apoptosis and enhancing innate anti-tumor immunity. Biochem. Pharmacol..

[121] Fischer K., Tognarelli S., Roesler S., Boedicker C., Schubert R., Steinle A., Klingebiel T., Bader P., Fulda S., Ullrich E. (2017). The Smac Mimetic BV6 Improves NK Cell-Mediated Killing of Rhabdomyosarcoma Cells by Simultaneously Targeting Tumor and Effector Cells. Front. Immunol..

[122] Brinkmann K., Hombach A., Seeger J.M., Wagner-Stippich D., Klubertz D., Kronke M., Abken H., Kashkar H. (2014). Second mitochondria-derived activator of caspase (SMAC) mimetic potentiates tumor susceptibility toward natural killer cell-mediated killing. Leuk. Lymphoma.

[123] Dougan S.K., Dougan M. (2018). Regulation of innate and adaptive antitumor immunity by IAP antagonists. Immunotherapy.

[124] Kearney C.J., Lalaoui N., Freeman A.J., Ramsbottom K.M., Silke J., Oliaro J. (2017). PD-L1 and IAPs co-operate to protect tumors from cytotoxic lymphocyte-derived TNF. Cell Death Differ..

[125] Runckel K., Barth M.J., Mavis C., Gu J.J., Hernandez-Ilizaliturri F.J. (2018). The SMAC mimetic LCL-161 displays antitumor activity in preclinical models of rituximab-resistant B-cell lymphoma. Blood Adv..

[126] Rettinger E., Glatthaar A., Abhari B.A., Oelsner S., Pfirrmann V., Huenecke S., Kuci S., Kreyenberg H., Willasch A.M., Klingebiel T. (2014). SMAC Mimetic BV6 Enables Sensitization of Resistant Tumor Cells but also Affects Cytokine-Induced Killer (CIK) Cells: A Potential Challenge for Combination Therapy. Front. Pediatrics.

[127] Beug S.T., Conrad D.P., Alain T., Korneluk R.G., Lacasse E.C. (2015). Combinatorial cancer immunotherapy strategies with proapoptotic small-molecule IAP antagonists. Int. J. Dev. Biol..

[128] Beug S.T., Beauregard C.E., Healy C., Sanda T., St-Jean M., Chabot J., Walker D.E., Mohan A., Earl N., Lun X. (2017). Smac mimetics synergize with immune checkpoint inhibitors to promote tumour immunity against glioblastoma. Nat. Commun..

[129] Shekhar T.M., Burvenich I.J.G., Harris M.A., Rigopoulos A., Zanker D., Spurling A., Parker B.S., Walkley C.R., Scott A.M., Hawkins C.J. (2019). Smac mimetics LCL161 and GDC-0152 inhibit osteosarcoma growth and metastasis in mice. BMC Cancer.

[130] Marquez M.E., Hernandez-Uzcategui O., Cornejo A., Vargas P., Da Costa O. (2015). Bone marrow stromal mesenchymal cells induce down regulation of CD20 expression on B-CLL: Implications for rituximab resistance in CLL. Br. J. Haematol..

[131] Dunn G.P., Old L.J., Schreiber R.D. (2004). The three Es of cancer immunoediting. Annu. Rev. Immunol..

[132] Vesely M.D., Kershaw M.H., Schreiber R.D., Smyth M.J. (2011). Natural innate and adaptive immunity to cancer. Annu. Rev. Immunol..

[133] Guerra N., Tan Y.X., Joncker N.T., Choy A., Gallardo F., Xiong N., Knoblaugh S., Cado D., Greenberg N.M., Raulet D.H. (2008). NKG2D-deficient mice are defective in tumor surveillance in models of spontaneous malignancy. Immunity.

[134] Iguchi-Manaka A., Kai H., Yamashita Y., Shibata K., Tahara-Hanaoka S., Honda S., Yasui T., Kikutani H., Shibuya K., Shibuya A. (2008). Accelerated tumor growth in mice deficient in DNAM-1 receptor. J. Exp. Med..

[135] Elboim M., Gazit R., Gur C., Ghadially H., Betser-Cohen G., Mandelboim O. (2010). Tumor immunoediting by NKp46. J. Immunol..

[136] O’Sullivan T., Saddawi-Konefka R., Vermi W., Koebel C.M., Arthur C., White J.M., Uppaluri R., Andrews D.M., Ngiow S.F., Teng M.W. (2012). Cancer immunoediting by the innate immune system in the absence of adaptive immunity. J. Exp. Med..

[137] Guillerey C., Smyth M.J. (2016). NK Cells and Cancer Immunoediting. Curr. Top. Microbiol. Immunol..

[138] Holdenrieder S., Stieber P., Peterfi A., Nagel D., Steinle A., Salih H.R. (2006). Soluble MICA in malignant diseases. Int. J. Cancer.

[139] Salih H.R., Rammensee H.G., Steinle A. (2002). Cutting edge: Down-regulation of MICA on human tumors by proteolytic shedding. J. Immunol..

[140] Chen Y., Lin W.S., Zhu W.F., Lin J., Zhou Z.F., Huang C.Z., Chen G., Shi Y., Guo Z.Q., Ye Y.B. (2016). Tumor MICA status predicts the efficacy of immunotherapy with cytokine-induced killer cells for patients with gastric cancer. Immunol. Res..

[141] Fan X.Y., Wang P.Y., Zhang C., Zhang Y.L., Fu Y., Zhang C., Li Q.X., Zhou J.N., Shan B.E., He D.W. (2017). All-trans retinoic acid enhances cytotoxicity of CIK cells against human lung adenocarcinoma by upregulating MICA and IL-2 secretion. Sci. Rep..

[142] Rautela J., Baschuk N., Slaney C.Y., Jayatilleke K.M., Xiao K., Bidwell B.N., Lucas E.C., Hawkins E.D., Lock P., Wong C.S. (2015). Loss of Host Type-I IFN Signaling Accelerates Metastasis and Impairs NK-cell Antitumor Function in Multiple Models of Breast Cancer. Cancer Immunol. Res..

[143] Freeman A.J., Vervoort S.J., Ramsbottom K.M., Kelly M.J., Michie J., Pijpers L., Johnstone R.W., Kearney C.J., Oliaro J. (2019). Natural Killer Cells Suppress T Cell-Associated Tumor Immune Evasion. Cell Rep..

[144] Guillerey C., Huntington N.D., Smyth M.J. (2016). Targeting natural killer cells in cancer immunotherapy. Nat. Immunol..

[145] Grossenbacher S.K., Canter R.J., Murphy W.J. (2016). Natural killer cell immunotherapy to target stem-like tumor cells. J. Immunother. Cancer.

[146] Tallerico R., Todaro M., Di Franco S., Maccalli C., Garofalo C., Sottile R., Palmieri C., Tirinato L., Pangigadde P.N., La Rocca R. (2013). Human NK cells selective targeting of colon cancer-initiating cells: A role for natural cytotoxicity receptors and MHC class I molecules. J. Immunol..

[147] Castriconi R., Daga A., Dondero A., Zona G., Poliani P.L., Melotti A., Griffero F., Marubbi D., Spaziante R., Bellora F. (2009). NK cells recognize and kill human glioblastoma cells with stem cell-like properties. J. Immunol..

[148] Pietra G., Manzini C., Vitale M., Balsamo M., Ognio E., Boitano M., Queirolo P., Moretta L., Mingari M.C. (2009). Natural killer cells kill human melanoma cells with characteristics of cancer stem cells. Int. Immunol..

[149] Patel S.A., Meyer J.R., Greco S.J., Corcoran K.E., Bryan M., Rameshwar P. (2010). Mesenchymal stem cells protect breast cancer cells through regulatory T cells: Role of mesenchymal stem cell-derived TGF-beta. J. Immunol..

[150] Kozlowska A.K., Tseng H.C., Kaur K., Topchyan P., Inagaki A., Bui V.T., Kasahara N., Cacalano N., Jewett A. (2016). Resistance to cytotoxicity and sustained release of interleukin-6 and interleukin-8 in the presence of decreased interferon-gamma after differentiation of glioblastoma by human natural killer cells. Cancer Immunol. Immunother..

[151] Garrido F. (2019). MHC/HLA Class I Loss in Cancer Cells. Adv. Exp. Med. Biol..

[152] Zeestraten E.C., Reimers M.S., Saadatmand S., Goossens-Beumer I.J., Dekker J.W., Liefers G.J., van den Elsen P.J., van de Velde C.J., Kuppen P.J. (2014). Combined analysis of HLA class I, HLA-E and HLA-G predicts prognosis in colon cancer patients. Br. J. Cancer.

[153] Nguyen S., Beziat V., Dhedin N., Kuentz M., Vernant J.P., Debre P., Vieillard V. (2009). HLA-E upregulation on IFN-gamma-activated AML blasts impairs CD94/NKG2A-dependent NK cytolysis after haplo-mismatched hematopoietic SCT. Bone Marrow Transplant..

[154] Morandi F., Pistoia V. (2014). Interactions between HLA-G and HLA-E in Physiological and Pathological Conditions. Front. Immunol..

[155] Nguyen S., Dhedin N., Vernant J.P., Kuentz M., Al Jijakli A., Rouas-Freiss N., Carosella E.D., Boudifa A., Debre P., Vieillard V. (2005). NK-cell reconstitution after haploidentical hematopoietic stem-cell transplantations: Immaturity of NK cells and inhibitory effect of NKG2A override GvL effect. Blood.

[156] Ruggeri L., Urbani E., Andre P., Mancusi A., Tosti A., Topini F., Blery M., Animobono L., Romagne F., Wagtmann N. (2016). Effects of anti-NKG2A antibody administration on leukemia and normal hematopoietic cells. Haematologica.

[157] Czuczman M.S., Olejniczak S., Gowda A., Kotowski A., Binder A., Kaur H., Knight J., Starostik P., Deans J., Hernandez-Ilizaliturri F.J. (2008). Acquirement of rituximab resistance in lymphoma cell lines is associated with both global CD20 gene and protein down-regulation regulated at the pretranscriptional and posttranscriptional levels. Clin. Cancer Res..

[158] Hiraga J., Tomita A., Sugimoto T., Shimada K., Ito M., Nakamura S., Kiyoi H., Kinoshita T., Naoe T. (2009). Down-regulation of CD20 expression in B-cell lymphoma cells after treatment with rituximab-containing combination chemotherapies: Its prevalence and clinical significance. Blood.

[159] Terui Y., Mishima Y., Sugimura N., Kojima K., Sakurai T., Mishima Y., Kuniyoshi R., Taniyama A., Yokoyama M., Sakajiri S. (2009). Identification of CD20 C-terminal deletion mutations associated with loss of CD20 expression in non-Hodgkin’s lymphoma. Clin. Cancer Res..

[160] Beers S.A., French R.R., Chan H.T., Lim S.H., Jarrett T.C., Vidal R.M., Wijayaweera S.S., Dixon S.V., Kim H., Cox K.L. (2010). Antigenic modulation limits the efficacy of anti-CD20 antibodies: Implications for antibody selection. Blood.

[161] Winiarska M., Bil J., Wilczek E., Wilczynski G.M., Lekka M., Engelberts P.J., Mackus W.J., Gorska E., Bojarski L., Stoklosa T. (2008). Statins impair antitumor effects of rituximab by inducing conformational changes of CD20. PLoS Med..

[162] Battke C., Ruiss R., Welsch U., Wimberger P., Lang S., Jochum S., Zeidler R. (2011). Tumour exosomes inhibit binding of tumour-reactive antibodies to tumour cells and reduce ADCC. Cancer Immunol. Immunother..

[163] Pedersen A.E., Jungersen M.B., Pedersen C.D. (2011). Monocytes mediate shaving of B-cell-bound anti-CD20 antibodies. Immunology.

[164] Sperinde J., Jin X., Banerjee J., Penuel E., Saha A., Diedrich G., Huang W., Leitzel K., Weidler J., Ali S.M. (2010). Quantitation of p95HER2 in paraffin sections by using a p95-specific antibody and correlation with outcome in a cohort of trastuzumab-treated breast cancer patients. Clin. Cancer Res..

[165] Scaltriti M., Rojo F., Ocana A., Anido J., Guzman M., Cortes J., Di Cosimo S., Matias-Guiu X., Ramon y Cajal S., Arribas J. (2007). Expression of p95HER2, a truncated form of the HER2 receptor, and response to anti-HER2 therapies in breast cancer. J. Natl. Cancer Inst..

[166] O’Donnell J.S., Teng M.W.L., Smyth M.J. (2019). Cancer immunoediting and resistance to T cell-based immunotherapy. Nat. Rev. Clin. Oncol..

[167] Mamessier E., Sylvain A., Thibult M.L., Houvenaeghel G., Jacquemier J., Castellano R., Goncalves A., Andre P., Romagne F., Thibault G. (2011). Human breast cancer cells enhance self tolerance by promoting evasion from NK cell antitumor immunity. J. Clin. Investig..

[168] Platonova S., Cherfils-Vicini J., Damotte D., Crozet L., Vieillard V., Validire P., Andre P., Dieu-Nosjean M.C., Alifano M., Regnard J.F. (2011). Profound coordinated alterations of intratumoral NK cell phenotype and function in lung carcinoma. Cancer Res..

[169] Halama N., Braun M., Kahlert C., Spille A., Quack C., Rahbari N., Koch M., Weitz J., Kloor M., Zoernig I. (2011). Natural killer cells are scarce in colorectal carcinoma tissue despite high levels of chemokines and cytokines. Clin. Cancer Res..

[170] Pesce S., Tabellini G., Cantoni C., Patrizi O., Coltrini D., Rampinelli F., Matta J., Vivier E., Moretta A., Parolini S. (2015). B7-H6-mediated downregulation of NKp30 in NK cells contributes to ovarian carcinoma immune escape. Oncoimmunology.

[171] Han B., Mao F.Y., Zhao Y.L., Lv Y.P., Teng Y.S., Duan M., Chen W., Cheng P., Wang T.T., Liang Z.Y. (2018). Altered NKp30, NKp46, NKG2D, and DNAM-1 Expression on Circulating NK Cells Is Associated with Tumor Progression in Human Gastric Cancer. J. Immunol. Res..

[172] Sanchez C.J., Le Treut T., Boehrer A., Knoblauch B., Imbert J., Olive D., Costello R.T. (2011). Natural killer cells and malignant haemopathies: A model for the interaction of cancer with innate immunity. Cancer Immunol. Immunother..

[173] Garcia-Iglesias T., Del Toro-Arreola A., Albarran-Somoza B., Del Toro-Arreola S., Sanchez-Hernandez P.E., Ramirez-Duenas M.G., Balderas-Pena L.M., Bravo-Cuellar A., Ortiz-Lazareno P.C., Daneri-Navarro A. (2009). Low NKp30, NKp46 and NKG2D expression and reduced cytotoxic activity on NK cells in cervical cancer and precursor lesions. BMC Cancer.

[174] Fregni G., Perier A., Pittari G., Jacobelli S., Sastre X., Gervois N., Allard M., Bercovici N., Avril M.F., Caignard A. (2011). Unique functional status of natural killer cells in metastatic stage IV melanoma patients and its modulation by chemotherapy. Clin. Cancer Res..

[175] Zaiatz-Bittencourt V., Finlay D.K., Gardiner C.M. (2018). Canonical TGF-beta Signaling Pathway Represses Human NK Cell Metabolism. J. Immunol..

[176] Castriconi R., Cantoni C., Della Chiesa M., Vitale M., Marcenaro E., Conte R., Biassoni R., Bottino C., Moretta L., Moretta A. (2003). Transforming growth factor beta 1 inhibits expression of NKp30 and NKG2D receptors: Consequences for the NK-mediated killing of dendritic cells. Proc. Natl. Acad. Sci. USA.

[177] Park A., Lee Y., Kim M.S., Kang Y.J., Park Y.J., Jung H., Kim T.D., Lee H.G., Choi I., Yoon S.R. (2018). Prostaglandin E2 Secreted by Thyroid Cancer Cells Contributes to Immune Escape Through the Suppression of Natural Killer (NK) Cell Cytotoxicity and NK Cell Differentiation. Front. Immunol..

[178] Balsamo M., Scordamaglia F., Pietra G., Manzini C., Cantoni C., Boitano M., Queirolo P., Vermi W., Facchetti F., Moretta A. (2009). Melanoma-associated fibroblasts modulate NK cell phenotype and antitumor cytotoxicity. Proc. Natl. Acad. Sci. USA.

[179] Della Chiesa M., Carlomagno S., Frumento G., Balsamo M., Cantoni C., Conte R., Moretta L., Moretta A., Vitale M. (2006). The tryptophan catabolite L-kynurenine inhibits the surface expression of NKp46- and NKG2D-activating receptors and regulates NK-cell function. Blood.

[180] Gaggero S., Bruschi M., Petretto A., Parodi M., Del Zotto G., Lavarello C., Prato C., Santucci L., Barbuto A., Bottino C. (2018). Nidogen-1 is a novel extracellular ligand for the NKp44 activating receptor. Oncoimmunology.

[181] Wang W., Guo H., Geng J., Zheng X., Wei H., Sun R., Tian Z. (2014). Tumor-released Galectin-3, a soluble inhibitory ligand of human NKp30, plays an important role in tumor escape from NK cell attack. J. Biol. Chem..

[182] de Andrade L.F., Tay R.E., Pan D., Luoma A.M., Ito Y., Badrinath S., Tsoucas D., Franz B., May K.F., Harvey C.J. (2018). Antibody-mediated inhibition of MICA and MICB shedding promotes NK cell-driven tumor immunity. Science.

[183] Schlecker E., Fiegler N., Arnold A., Altevogt P., Rose-John S., Moldenhauer G., Sucker A., Paschen A., von Strandmann E.P., Textor S. (2014). Metalloprotease-mediated tumor cell shedding of B7-H6, the ligand of the natural killer cell-activating receptor NKp30. Cancer Res..

[184] MacFarlane A.W.t., Jillab M., Plimack E.R., Hudes G.R., Uzzo R.G., Litwin S., Dulaimi E., Al-Saleem T., Campbell K.S. (2014). PD-1 expression on peripheral blood cells increases with stage in renal cell carcinoma patients and is rapidly reduced after surgical tumor resection. Cancer Immunol. Res..

[185] Pesce S., Greppi M., Tabellini G., Rampinelli F., Parolini S., Olive D., Moretta L., Moretta A., Marcenaro E. (2017). Identification of a subset of human natural killer cells expressing high levels of programmed death 1: A phenotypic and functional characterization. J. Allergy Clin. Immunol..

[186] Merino A., Zhang B., Dougherty P., Luo X., Wang J., Blazar B.R., Miller J.S., Cichocki F. (2019). Chronic stimulation drives human NK cell dysfunction and epigenetic reprograming. J. Clin. Investig..

[187] da Silva I.P., Gallois A., Jimenez-Baranda S., Khan S., Anderson A.C., Kuchroo V.K., Osman I., Bhardwaj N. (2014). Reversal of NK-cell exhaustion in advanced melanoma by Tim-3 blockade. Cancer Immunol. Res..

[188] Zhang Q., Bi J., Zheng X., Chen Y., Wang H., Wu W., Wang Z., Wu Q., Peng H., Wei H. (2018). Blockade of the checkpoint receptor TIGIT prevents NK cell exhaustion and elicits potent anti-tumor immunity. Nat. Immunol..

[189] Putz E.M., Guillerey C., Kos K., Stannard K., Miles K., Delconte R.B., Takeda K., Nicholson S.E., Huntington N.D., Smyth M.J. (2017). Targeting cytokine signaling checkpoint CIS activates NK cells to protect from tumor initiation and metastasis. Oncoimmunology.

[190] Molgora M., Bonavita E., Ponzetta A., Riva F., Barbagallo M., Jaillon S., Popovic B., Bernardini G., Magrini E., Gianni F. (2017). IL-1R8 is a checkpoint in NK cells regulating anti-tumour and anti-viral activity. Nature.

[191] Vitale M., Cantoni C., Pietra G., Mingari M.C., Moretta L. (2014). Effect of tumor cells and tumor microenvironment on NK-cell function. Eur. J. Immunol..

[192] Terren I., Orrantia A., Vitalle J., Zenarruzabeitia O., Borrego F. (2019). NK Cell Metabolism and Tumor Microenvironment. Front. Immunol..

[193] Velasquez S.Y., Killian D., Schulte J., Sticht C., Thiel M., Lindner H.A. (2016). Short Term Hypoxia Synergizes with Interleukin 15 Priming in Driving Glycolytic Gene Transcription and Supports Human Natural Killer Cell Activities. J. Biol. Chem..

[194] Balsamo M., Manzini C., Pietra G., Raggi F., Blengio F., Mingari M.C., Varesio L., Moretta L., Bosco M.C., Vitale M. (2013). Hypoxia downregulates the expression of activating receptors involved in NK-cell-mediated target cell killing without affecting ADCC. Eur. J. Immunol..

[195] Parodi M., Raggi F., Cangelosi D., Manzini C., Balsamo M., Blengio F., Eva A., Varesio L., Pietra G., Moretta L. (2018). Hypoxia Modifies the Transcriptome of Human NK Cells, Modulates Their Immunoregulatory Profile, and Influences NK Cell Subset Migration. Front. Immunol..

[196] Terry S., Abdou A., Engelsen A.S.T., Buart S., Dessen P., Corgnac S., Collares D., Meurice G., Gausdal G., Baud V. (2019). AXL Targeting Overcomes Human Lung Cancer Cell Resistance to NK- and CTL-Mediated Cytotoxicity. Cancer Immunol. Res..

[197] Noman M.Z., Desantis G., Janji B., Hasmim M., Karray S., Dessen P., Bronte V., Chouaib S. (2014). PD-L1 is a novel direct target of HIF-1alpha, and its blockade under hypoxia enhanced MDSC-mediated T cell activation. J. Exp. Med..

[198] Noman M.Z., Chouaib S. (2014). Targeting hypoxia at the forefront of anticancer immune responses. Oncoimmunology.

[199] Krzywinska E., Kantari-Mimoun C., Kerdiles Y., Sobecki M., Isagawa T., Gotthardt D., Castells M., Haubold J., Millien C., Viel T. (2017). Loss of HIF-1alpha in natural killer cells inhibits tumour growth by stimulating non-productive angiogenesis. Nat. Commun..

[200] Muz B., de la Puente P., Azab F., Luderer M., Azab A.K. (2014). The role of hypoxia and exploitation of the hypoxic environment in hematologic malignancies. Mol. Cancer Res..

[201] Cantoni C., Huergo-Zapico L., Parodi M., Pedrazzi M., Mingari M.C., Moretta A., Sparatore B., Gonzalez S., Olive D., Bottino C. (2016). NK Cells, Tumor Cell Transition, and Tumor Progression in Solid Malignancies: New Hints for NK-Based Immunotherapy?. J. Immunol. Res..

[202] Carrega P., Bonaccorsi I., Di Carlo E., Morandi B., Paul P., Rizzello V., Cipollone G., Navarra G., Mingari M.C., Moretta L. (2014). CD56(bright)perforin(low) noncytotoxic human NK cells are abundant in both healthy and neoplastic solid tissues and recirculate to secondary lymphoid organs via afferent lymph. J. Immunol..

[203] Campbell J.J., Qin S., Unutmaz D., Soler D., Murphy K.E., Hodge M.R., Wu L., Butcher E.C. (2001). Unique subpopulations of CD56+ NK and NK-T peripheral blood lymphocytes identified by chemokine receptor expression repertoire. J. Immunol..

[204] Meng W., Xue S., Chen Y. (2018). The role of CXCL12 in tumor microenvironment. Gene.

[205] Tutunea-Fatan E., Majumder M., Xin X., Lala P.K. (2015). The role of CCL21/CCR7 chemokine axis in breast cancer-induced lymphangiogenesis. Mol. Cancer.

[206] Sand L.G., Berghuis D., Szuhai K., Hogendoorn P.C. (2016). Expression of CCL21 in Ewing sarcoma shows an inverse correlation with metastases and is a candidate target for immunotherapy. Cancer Immunol. Immunother..

[207] Castriconi R., Dondero A., Bellora F., Moretta L., Castellano A., Locatelli F., Corrias M.V., Moretta A., Bottino C. (2013). Neuroblastoma-derived TGF-beta1 modulates the chemokine receptor repertoire of human resting NK cells. J. Immunol..

[208] Costa D., Vene R., Benelli R., Romairone E., Scabini S., Catellani S., Rebesco B., Mastracci L., Grillo F., Minghelli S. (2018). Targeting the Epidermal Growth Factor Receptor Can Counteract the Inhibition of Natural Killer Cell Function Exerted by Colorectal Tumor-Associated Fibroblasts. Front. Immunol..

[209] Trotta R., Dal Col J., Yu J., Ciarlariello D., Thomas B., Zhang X., Allard J., Wei M., Mao H., Byrd J.C. (2008). TGF-beta utilizes SMAD3 to inhibit CD16-mediated IFN-gamma production and antibody-dependent cellular cytotoxicity in human NK cells. J. Immunol..

[210] Watanabe M., Kono K., Kawaguchi Y., Mizukami Y., Mimura K., Maruyama T., Izawa S., Fujii H. (2010). NK cell dysfunction with down-regulated CD16 and up-regulated CD56 molecules in patients with esophageal squamous cell carcinoma. Dis. Esophagus.

[211] Kawaguchi Y., Kono K., Mimura K., Sugai H., Akaike H., Fujii H. (2007). Cetuximab induce antibody-dependent cellular cytotoxicity against EGFR-expressing esophageal squamous cell carcinoma. Int. J. Cancer.

[212] Bedi A., Chang X., Noonan K., Pham V., Bedi R., Fertig E.J., Considine M., Califano J.A., Borrello I., Chung C.H. (2012). Inhibition of TGF-beta enhances the in vivo antitumor efficacy of EGF receptor-targeted therapy. Mol. Cancer Ther..

[213] Wang S.Y., Veeramani S., Racila E., Cagley J., Fritzinger D.C., Vogel C.W., St John W., Weiner G.J. (2009). Depletion of the C3 component of complement enhances the ability of rituximab-coated target cells to activate human NK cells and improves the efficacy of monoclonal antibody therapy in an in vivo model. Blood.

[214] Battella S., Cox M.C., Santoni A., Palmieri G. (2016). Natural killer (NK) cells and anti-tumor therapeutic mAb: Unexplored interactions. J. Leukoc. Biol..

[215] Wu J., Mishra H.K., Walcheck B. (2019). Role of ADAM17 as a regulatory checkpoint of CD16A in NK cells and as a potential target for cancer immunotherapy. J. Leukoc. Biol..

[216] Balsamo M., Vermi W., Parodi M., Pietra G., Manzini C., Queirolo P., Lonardi S., Augugliaro R., Moretta A., Facchetti F. (2012). Melanoma cells become resistant to NK-cell-mediated killing when exposed to NK-cell numbers compatible with NK-cell infiltration in the tumor. Eur. J. Immunol..

[217] Andre P., Denis C., Soulas C., Bourbon-Caillet C., Lopez J., Arnoux T., Blery M., Bonnafous C., Gauthier L., Morel A. (2018). Anti-NKG2A mAb Is a Checkpoint Inhibitor that Promotes Anti-tumor Immunity by Unleashing Both T and NK Cells. Cell.

[218] Gauthier L., Morel A., Anceriz N., Rossi B., Blanchard-Alvarez A., Grondin G., Trichard S., Cesari C., Sapet M., Bosco F. (2019). Multifunctional Natural Killer Cell Engagers Targeting NKp46 Trigger Protective Tumor Immunity. Cell.

[219] Sarhan D., Brandt L., Felices M., Guldevall K., Lenvik T., Hinderlie P., Curtsinger J., Warlick E., Spellman S.R., Blazar B.R. (2018). 161533 TriKE stimulates NK-cell function to overcome myeloid-derived suppressor cells in MDS. Blood Adv..

[220] Tumino N., Besi F., Di Pace A.L., Mariotti F.R., Merli P., Li Pira G., Galaverna F., Pitisci A., Ingegnere T., Pelosi A. (2019). PMN-MDSC are a new target to rescue graft-versus-leukemia activity of NK cells in haplo-HSC transplantation. Leukemia.

[221] Khan K.D., Emmanouilides C., Benson D.M., Hurst D., Garcia P., Michelson G., Milan S., Ferketich A.K., Piro L., Leonard J.P. (2006). A phase 2 study of rituximab in combination with recombinant interleukin-2 for rituximab-refractory indolent non-Hodgkin’s lymphoma. Clin. Cancer Res..

[222] Ahmadzadeh M., Rosenberg S.A. (2006). IL-2 administration increases CD4+ CD25(hi) Foxp3+ regulatory T cells in cancer patients. Blood.

[223] Jie H.B., Schuler P.J., Lee S.C., Srivastava R.M., Argiris A., Ferrone S., Whiteside T.L., Ferris R.L. (2015). CTLA-4(+) Regulatory T Cells Increased in Cetuximab-Treated Head and Neck Cancer Patients Suppress NK Cell Cytotoxicity and Correlate with Poor Prognosis. Cancer Res..

[224] Julia E.P., Amante A., Pampena M.B., Mordoh J., Levy E.M. (2018). Avelumab, an IgG1 anti-PD-L1 Immune Checkpoint Inhibitor, Triggers NK Cell-Mediated Cytotoxicity and Cytokine Production Against Triple Negative Breast Cancer Cells. Front. Immunol..

[225] Datar I., Sanmamed M.F., Wang J., Henick B.S., Choi J., Badri T., Dong W., Mani N., Toki M., Mejias L.D. (2019). Expression Analysis and Significance of PD-1, LAG-3, and TIM-3 in Human Non-Small Cell Lung Cancer Using Spatially Resolved and Multiparametric Single-Cell Analysis. Clin. Cancer Res..

[226] Dong W., Wu X., Ma S., Wang Y., Nalin A.P., Zhu Z., Zhang J., Benson D.M., He K., Caligiuri M.A. (2019). The Mechanism of Anti-PD-L1 Antibody Efficacy against PD-L1-Negative Tumors Identifies NK Cells Expressing PD-L1 as a Cytolytic Effector. Cancer Discov..

[227] Hwang W.L., Pike L.R.G., Royce T.J., Mahal B.A., Loeffler J.S. (2018). Safety of combining radiotherapy with immune-checkpoint inhibition. Nat. Rev. Clin. Oncol..

[228] Roger A., Finet A., Boru B., Beauchet A., Mazeron J.J., Otzmeguine Y., Blom A., Longvert C., de Maleissye M.F., Fort M. (2018). Efficacy of combined hypo-fractionated radiotherapy and anti-PD-1 monotherapy in difficult-to-treat advanced melanoma patients. Oncoimmunology.

[229] Formenti S.C., Lee P., Adams S., Goldberg J.D., Li X., Xie M.W., Ratikan J.A., Felix C., Hwang L., Faull K.F. (2018). Focal Irradiation and Systemic TGFbeta Blockade in Metastatic Breast Cancer. Clin. Cancer Res..

[230] Aboudaram A., Modesto A., Chaltiel L., Gomez-Roca C., Boulinguez S., Sibaud V., Delord J.P., Chira C., Delannes M., Moyal E. (2017). Concurrent radiotherapy for patients with metastatic melanoma and receiving anti-programmed-death 1 therapy: A safe and effective combination. Melanoma Res..

[231] Sivori S., Meazza R., Quintarelli C., Carlomagno S., Della Chiesa M., Falco M., Moretta L., Locatelli F., Pende D. (2019). NK Cell-Based Immunotherapy for Hematological Malignancies. J. Clin. Med..

[232] Souza-Fonseca-Guimaraes F., Cursons J., Huntington N.D. (2019). The Emergence of Natural Killer Cells as a Major Target in Cancer Immunotherapy. Trends Immunol..

